# p53 Loss Mediates Hypersensitivity to ETS Transcription Factor Inhibition Based on PARylation-Mediated Cell Death Induction

**DOI:** 10.3390/cancers12113205

**Published:** 2020-10-30

**Authors:** Carina Dinhof, Christine Pirker, Philipp Kroiss, Dominik Kirchhofer, Lisa Gabler, Johannes Gojo, Daniela Lötsch-Gojo, Mirjana Stojanovic, Gerald Timelthaler, Franziska Ferk, Siegfried Knasmüller, Johannes Reisecker, Sabine Spiegl-Kreinecker, Peter Birner, Matthias Preusser, Walter Berger

**Affiliations:** 1 Department of Medicine I, Institute of Cancer Research, Medical University of Vienna, 1090 Vienna, Austria; carina.dinhof@meduniwien.ac.at (C.D.); christine.pirker@meduniwien.ac.at (C.P.); philippkroiss@outlook.at (P.K.); dominik.kirchhofer@meduniwien.ac.at (D.K.); lisa.gabler@meduniwien.ac.at (L.G.); daniela.loetsch-gojo@meduniwien.ac.at (D.L.-G.); mirjana.stojanovic@meduniwien.ac.at (M.S.); gerald.timelthaler@meduniwien.ac.at (G.T.); franziska.ferk@meduniwien.ac.at (F.F.); siegfried.knasmueller@meduniwien.ac.at (S.K.); johannes.reisecker@meduniwien.ac.at (J.R.); 2 Comprehensive Cancer Center, Medical University of Vienna, 1090 Vienna, Austria; johannes.gojo@meduniwien.ac.at; 3 Department of Pediatrics and Adolescent Medicine and Comprehensive Center for Pediatrics, Medical University of Vienna, 1090 Vienna, Austria; 4 Department of Neurosurgery, Medical University of Vienna, 1090 Vienna, Austria; 5 Department of Neurosurgery, Neuromed Campus, Kepler University Hospital GmbH, Johannes Kepler University, 4040 Linz, Austria; sabine.spiegl-kreinecker@kepleruniklinikum.at; 6 Clinical Institute of Pathology, Medical University of Vienna, 1090 Vienna, Austria; peter.birner@meduniwien.ac.at; 7 Division of Oncology, Department of Medicine I, Medical University of Vienna, 1090 Vienna, Austria; matthias.preusser@meduniwien.ac.at

**Keywords:** ETS1, p53, ETS factor inhibitor, YK-4-279, PARylation, parthanatos

## Abstract

**Simple Summary:**

ETS transcription factors are potent oncogenic drivers in several cancer types and represent promising therapeutic targets. However, molecular factors influencing response to ETS factor inhibition are widely unknown so far. Here, we uncover that sensitivity of cancer cells against ETS factor blockade by the small molecule inhibitor YK-4-279 is strongly promoted by p53 loss in a MAPK-driven background. Induction of a parthanatos-like cell death based on a deregulated MAPK/ETS1/p53/PARP1 signal axis is identified as underlying molecular mechanism. Hence, this study suggests a novel and biomarker-driven therapeutic strategy for p53-deleted tumours, generally known for their profound therapy resistance.

**Abstract:**

The small-molecule E26 transformation-specific (ETS) factor inhibitor YK-4-279 was developed for therapy of ETS/EWS fusion-driven Ewing’s sarcoma. Here we aimed to identify molecular factors underlying YK-4-279 responsiveness in ETS fusion-negative cancers. Cell viability screenings that deletion of *P53* induced hypersensitization against YK-4-279 especially in the BRAF^V600E^-mutated colon cancer model RKO. This effect was comparably minor in the BRAF wild-type HCT116 colon cancer model. Out of all ETS transcription factor family members, especially ETS1 overexpression at mRNA and protein level was induced by deletion of *P53* specifically under BRAF-mutated conditions. Exposure to YK-4-279 reverted ETS1 upregulation induced by *P53* knock-out in RKO cells. Despite upregulation of p53 by YK-4-279 itself in RKOp53 wild-type cells, YK-4-279-mediated hyperphosphorylation of histone histone H2A.x was distinctly more pronounced in the *P53* knock-out background. YK-4-279-induced cell death in RKOp53-knock-out cells involved hyperPARylation of PARP1, translocation of the apoptosis-inducible factor AIF into nuclei, and induction of mitochondrial membrane depolarization, all hallmarks of parthanatos. Accordingly, pharmacological PARP as well as BRAF^V600E^ inhibition showed antagonistic activity with YK-4-279 especially in the *P53* knock-out background. Taken together, we identified ETS factor inhibition as a promising strategy for the treatment of notoriously therapy-resistant p53-null solid tumours with activating MAPK mutations.

## 1. Introduction

The E26 transformation-specific (ETS) transcription factor family comprises 28 members, grouped into various subfamilies [1,2,3,4]. Their functions are multifaceted, ranging from the regulation of apoptosis, differentiation, proliferation, and angiogenesis to tissue remodeling [5,6]. Aberrant ETS signaling is mainly caused by chromosomal translocations in hematological malignancies, but also in solid tumors including Ewing’s Sarcomas (ES) and prostate cancer [1,5,6]. ES is driven by a chromosomal translocation of the EWS-coding EWS RNA-binding protein 1 (*EWSR1*) gene to an ETS factor gene, which is friend leukaemia integration 1 transcription factor (*FLI1*) in the majority of cases, generating an oncogenic fusion protein [7]. Consequently, multiple attempts were made to target this oncogenic driver for precision therapy of ES. The small molecular drug YK-4-279 has been proposed to block the interaction of RNA Helicase A (RHA) with the EWS/ETS fusion protein, crucial for the oncogenic transcriptional activity [8,9]. Efficacy against ES as well as ETS fusion-positive prostate cancer and SPIB- or SPI1-driven B-cell lymphoma models has been demonstrated in vitro and in vivo [10,11,12,13,14]. Currently, a derivative of YK-4-279 called TK216 is being tested in patients with relapsed and refractory ES in a clinical phase 1 study (NCT02657005). However, YK-4-279 activity might not be restricted to tumors driven by ETS fusion oncogenes, but may also inhibit tumor-promoting signals derived from overexpressed wild-type ETS factors. Thus, we have previously shown that brain tumors with enhanced telomerase activity, resulting from telomerase reverse transcriptase (*TERT*) promoter mutations, which generate de novo ETS factor binding sites, were more susceptible to YK-4-279 [15,16]. However, the precise molecular determinants underlying the anti-cancer activity of YK-4-279 are not entirely dissected so far and even ETS-independent targets have been proposed [17].

Here we aimed to identify molecular factors determining YK-4-279 responsiveness, focusing on ETS fusion-negative cancer models with or without intact p53 signaling. We describe parthanatos-like cell death induction by YK-4-279 specifically in notoriously therapy-resistant p53-null solid tumor cells driven by BRAF^V600E^/MAPK hyperactivation and dissect the underlying signaling mechanisms. 

## 2. Results

### 2.1. p53 Loss Renders Cells Hypersensitive against YK-4-279

**Figure 1:**
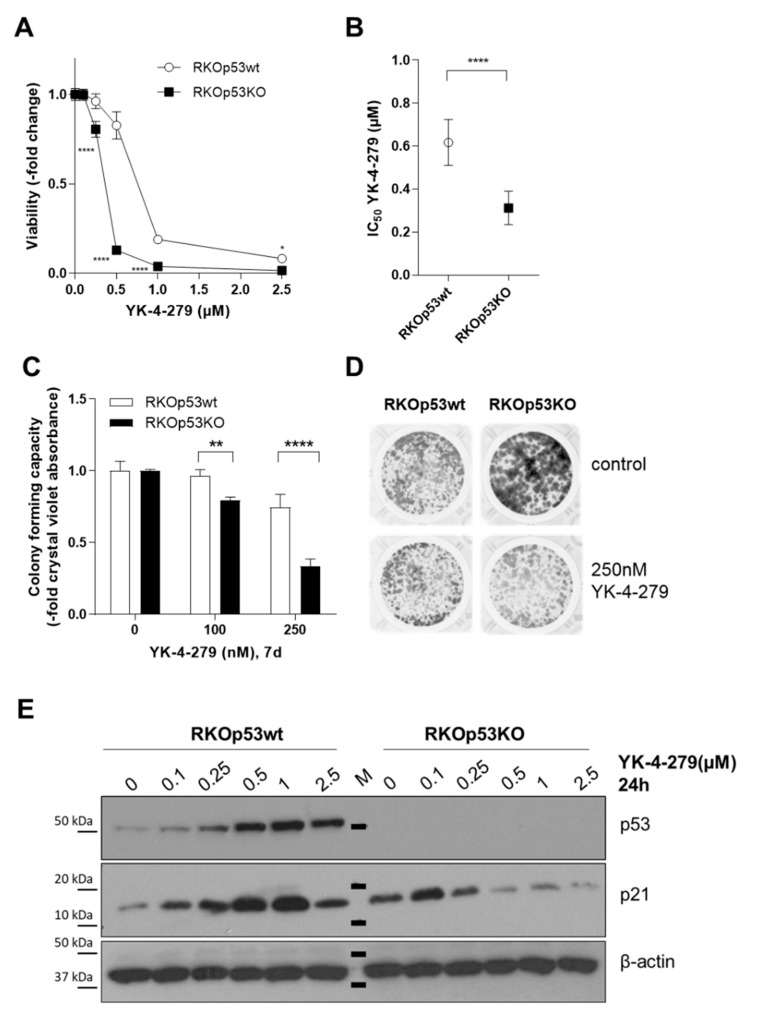
Loss of p53 mediates hypersensitivity to YK-4-279. (**A**) Impact of 72 h exposure to YK-4-279 at the indicated concentrations on viability of RKO colon carcinoma cells with wild-type (RKOp53wt) or deleted p53 (RKOp53KO) background was determined by 3-(4,5-dimethylthiazol-2-yl)-2,5-diphenyltetrazolium (MTT)-based survival assay. Values are given as mean ± standard deviations (SDs) of one representative experiment performed in triplicate. Statistical comparison was performed by two-way ANOVA with Bonferroni post-test. (**B**) Mean IC_50_ values for RKOp53wt and RKOp53KO cells derived from at least 4 independent experiments are given in µM ± SD. One-way ANOVA was used to test for statistical significance. (**C**) Colony formation capacity of RKOp53wt and RKOp53KO cells treated for 7 days with YK-4-279 at the indicated concentrations. Results are based on quantification of crystal violet staining (fluorescence intensity). Values are given normalized to untreated controls as means ± SD of one representative out of three experiments performed in triplicate. Statistical analysis was performed by two-way-ANOVA with Bonferroni post-test. (**D**) Representative photographs for the experiment analyzed in (**C**) are shown. (**E**) Protein expression levels of p53 and p21 following YK-4-279 treatment as indicated in the two RKO sublines were analyzed by Western blot. β-actin served as loading control. M = marker. Concerning all statistical analyses, p values less than 0.05 were considered significant, with * p < 0.05, ** p < 0.01, **** p < 0.0001.

In order to assess a possible connection between p53 and responsiveness to the small-molecule inhibitor YK-4-279, we analyzed a panel of different cancer cell lines (colon cancer, ES, melanoma) with known p53 wild-type (n = 5) or loss-of-function (n = 5) background. Strikingly, in all tested cancer models, p53 loss of function was associated with hypersensitivity against YK-4-279 (Figure 1A,B, and Supplementary Figure S1). Especially in the *P53* knock-out subclone of the BRAF^V600E^-mutated colon carcinoma model RKO (RKOp53KO), the ETS factor inhibitor was already active in a nanomolar range (Figure 1A,B), while the effect was distinctly weaker in the BRAF wild-type HCT116 colon cancer model (Supplementary Figure S1C,D). Additionally, in the case of ES, the *TP53*-mutated RD-ES cell model was explicitly more responsive against YK-4-279 as compared to the p53 wild-type FP-BH cells (Figure S1A). This relationship was additionally confirmed in a panel of melanoma cells (p53/p21-responsive n = 2, p53/p21-non-responsive n = 2) (Supplementary Figure S1B) [18]. Additionally, the impact of YK-4-279 on the clonogenic potential of the RKO model was tested by long-term exposure assays. Again, RKOp53KO cells were significantly more susceptible to ETS factor inhibition than RKOp53wt cells from as low as 100 nM onwards (Figure 1C,D). In the p53 wild-type clone, YK-4-279 triggered a functional p53 response accompanied by downstream p21 activation, while p21 expression in RKOp53KO cells was even reduced at higher YK-4-279 concentrations (Figure 1E).

### 2.2. Loss of p53 Causes ETS1 Overexpression

**Figure 2:**
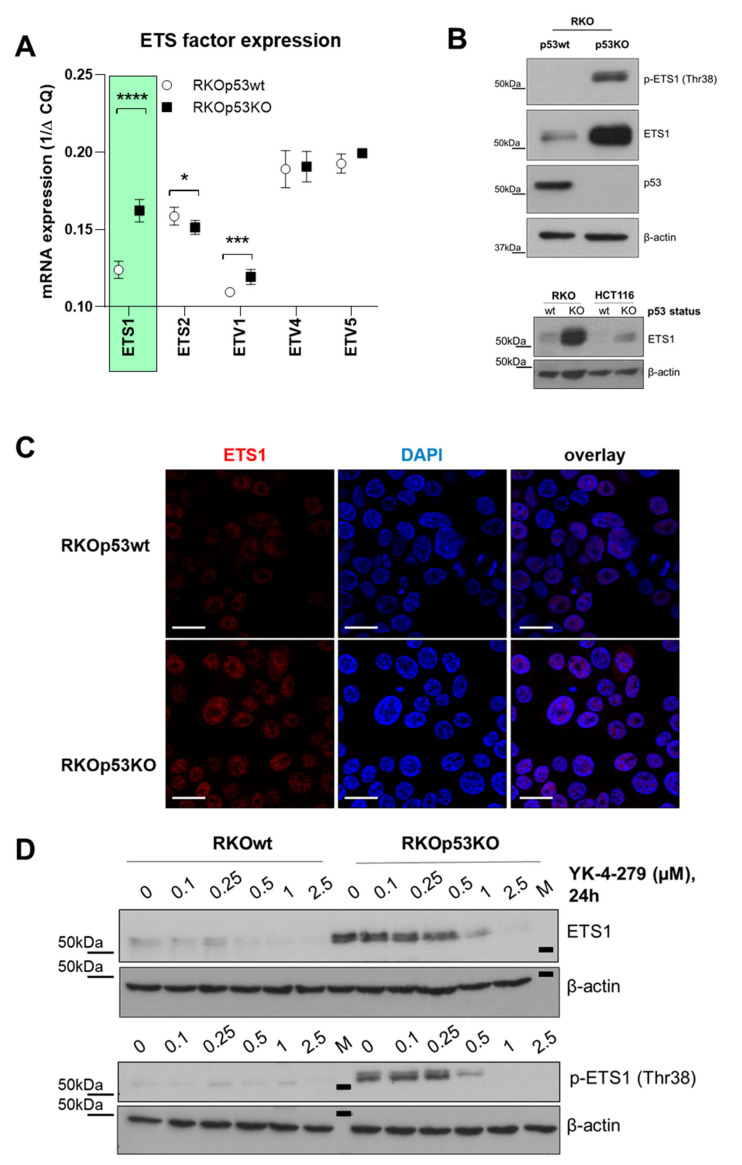
Expression/phosphorylation of ETS1 is increased in a p53 knock-out RKO colon cancer background. (**A**) mRNA expression levels of the indicated E26 transformation-specific ETS factors were assessed by qRT-PCR in the RKO cell model as indicated. Values were normalized to the housekeeping gene *RPL-41*. Students *t*-test was used for testing statistical significance with * p < 0.05, *** p < 0.001, and **** p < 0.0001. (**B**) Expression of ETS1 in the indicated colon cancer cell models was detected by Western blot analysis of total protein extracts. In the upper panel, p53 expression levels are shown for background confirmation. (**C**) Subcellular localization of ETS1 (red) in RKOp53wt and RKOp53KO cells was determined by immunofluorescence staining. 4′,6-Diamidino-2-phenylindole DAPI (blue) was used as nuclear counterstain. Representative fluorescence photomicrographs are shown. The scale bar indicates 20 µm. (**D**) Impact of YK-4-279 on expression of total and phosphorylated (Thr38) ETS1 levels in RKOp53wt and RKOp53KO cells was detected by Western blot analysis. β-actin was used as loading control.

Next, we investigated factors underlying p53 loss-mediated YK-4-279 hypersensitivity by analyzing the mRNA expression of ETS transcription factor genes in the RKO model. Expression of only 4 out of 24 ETS factor genes was more than two times upregulated in the RKOp53KO subline, namely *ELK3*, *ETS1*, *ELF1,* and *SPIB* (Supplementary Figure S2). Out of these, *ETS1* has especially been reported to interact with p53-mediated signaling [18,19,20,21]. *ETS1* mRNA upregulation in the RKOp53KO model was additionally confirmed by qRT-PCR (4.7-fold upregulation as compared to the p53wt subclone; Figure 2A). Enhanced mRNA levels translated well into distinctly higher amounts of total and activated (Thr38 phosphorylated) ETS1 proteins in the RKOp53KO background (Figure 2B, upper panel). Interestingly, p53 loss caused massive ETS1 overexpression solely in the BRAF mutant RKO but only weak upregulation in the BRAF wild-type HCT116 cell model (Figure 2B, lower panel), paralleling YK-4-279 responsiveness. Clearly enhanced ETS1 activation in RKOp53KO cells became further visible by immunofluorescence staining, demonstrating enhanced ETS1 accumulation in the nucleus (Figure 2C). Apart from this, total and phosphorylated ETS1 declined dose-dependently upon application of YK-4-279 in RKOp53KO cells, whereas in RKOp53wt again only very minor amounts of ETS1 were detectable (Figure 2D). This implicates that, out of the upregulated ETS factors, ETS1 might play a central role in YK-4-279-mediated hypersensitivity of RKOp53KO cells. Considering that ETS1 is a major downstream effector of the MAPK pathway [22], the BRAF^V600E^ mutant and, hence, MAPK-driven background of the RKO model might further strengthen this assumption. Indeed, exposure to the BRAF inhibitor dabrafenib completely reversed ETS1 expression in both RKO sublines, proving that ETS1 overexpression in RKOp53KO cells relies on an active MAPK pathway (Supplementary Figure S3A). Accordingly, combination of the BRAF inhibitor dabrafenib and YK-4-279 in cell viability assays resulted in antagonistic effects specifically in RKOp53KO cells but not in RKOp53wt nor in both HCT116 sublines (Supplementary Figure S3B), which were all low in terms of ETS1 expression. Remarkably, RKOp53KO cells exhibited enhanced susceptibility to single-drug BRAF inhibition as compared to the RKOp53wt model (Supplementary Figure S3C), indicating enhanced MAPK pathway dependency induced by a *P53* deletion. 

### 2.3. ETS1 Plays a Key Role in p53 Loss-Mediated YK-4-279 Responsiveness

In order to test whether wild-type p53 indeed attenuates YK-4-279 responses via ETS1 downregulation, combination experiments with Nutlin-3, a small molecular inhibitor of MDM2 and thus a p53 stabilizer [23], were performed. In line with our hypothesis, application of Nutlin-3 induced downregulation of ETS1 selectively in the RKOp53wt cells (Supplementary Figure S4A) and melanoma models with intact p53/p21 responses (Supplementary Figure S4B), strongly supporting a direct connection between p53 status and ETS1 protein expression. For combination viability tests, as RKOp53wt cells intrinsically harbor only very low levels of ETS1, we employed the ETS1-positive melanoma models with either intact or disrupted p53/p21 response. As expected, p53/p21-responsive melanoma cells exhibited sensitivity against Nutlin-3 in a single-agent setting, while non-responsive cell lines remained unaffected (Supplementary Figure S5A). In the combination approach, Nutlin-3 treatment indeed significantly protected against the cytotoxic effect of YK-4-279 selectively in the cell models with a functional p53 response. In contrast, this effect was absent in the p53/p21 loss-of-function background melanomas, and in the VM47 model even an unexpected synergistic effect was observed (Supplementary Figure S5B). This finding might either reflect an ETS factor-dependent effect of Nutlin-3 on the specific non-functional p53 axis in VM47 melanoma cells or p53-unrelated off-target effects of Nutlin-3. In accordance with the literature [24], YK-4-279 and Nutlin-3 showed synergistic activity against the EWS/FLI1-driven Ewing sarcoma model FP-BH despite harboring wild-type p53 (Supplementary Figure S5C). This reflects a release of EWS/FLI1-mediated p53 suppression by YK-4-279, thus allowing efficient p53 activation by Nutlin-3 [24]. In contrast to Nutlin-3, YK-4-279 treatment resulted in reduced ETS1 protein levels in all melanoma models tested independent of p53/p21 response status (Supplementary Figure S5D).

**Figure 3:**
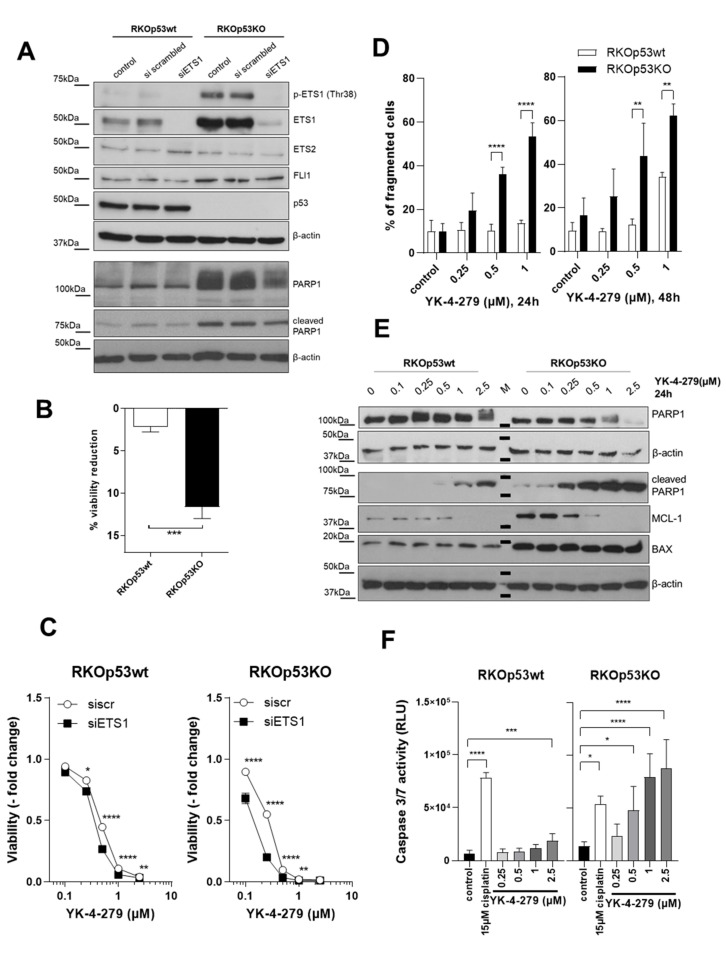
Impact of p53 status on YK-4-279-mediated cell death induction and role of ETS1. (**A**) Efficacy of ETS1 knock-down upon 72-h siRNA treatment and impact on the protein levels of the indicated ETS factors, p53, as well as total and cleaved PARP were determined by Western blot in the indicated RKO cell subclones. β-actin served as loading control. (**B**) Effect of ETS1 knock-down on RKO cell viability was measured 72 h post-transfection. Data are given as a percentage of viability reduction by ETS1-specific as compared to scrambled-control siRNA (mean ± SD) from three experiments. Statistical analysis was performed using unpaired Student’s *t*-test. (**C**) Effect of ETS1 knockdown (72 h) on sensitivity of the indicated RKO sublines against a 72-h treatment with YK-4-279 was assessed by 3-(4,5-dimethylthiazol-2-yl)-2,5-diphenyltetrazolium (MTT)-based viability assay. Statistical analysis was performed using two-way ANOVA with Bonferroni post-test. (**D**) Percentage of fragmented RKO cells following YK-4-279 exposure as indicated was determined by flow cytometry. Values are given as mean ± SD of three independent experiments. Statistical significance was tested by two-way-ANOVA with Bonferroni post-test. (**E**) Impact of a 24-h YK-4-279 exposure at the indicated concentrations on expression/cleavage of PARP1 as well as the bcl-2 family members MCL-1 (anti-apoptotic) and BAX (pro-apoptotic) was analyzed by Western blot. β-actin served as loading control. M = marker. (**F**) Effect of 24-h YK-4-279 exposure on caspase 3/7 activity in RKOp53wt as compared to RKOp53KO cells was determined by luminescence assay. Results are given as mean (± SD) relative luminescence units (RLUs) from three experiments in triplicate. One-way ANOVA with Bonferroni post-test was used for testing statistical significances. Concerning all statistical analyses, *p* values less than 0.05 were considered significant, with * *p* < 0.05, ** *p* < 0.01, *** *p* < 0.001, and **** *p* < 0.0001.

Next, we analyzed the impact of ETS1 loss on cell viability and YK-4-279 sensitivity in the RKO colon cancer model. Specific and persistent siRNA-mediated knock-down of ETS1 was proven by Western blot analysis, while ETS2 and FLI1 protein levels remained unaffected (Figure 3A). Interestingly, the *P53*-deleted RKO subline distinctly overexpressed the DNA repair protein and caspase substrate PARP1 [25]. ETS1 knock-down induced downregulation of total PARP1 expression, however, without enhancing classical apoptosis-associated PARP1 cleavage. Additionally, siRNA-mediated ETS1 knockdown led to a significantly decreased viability specifically in RKOp53KO cells (Figure 3B). Moreover, sensitivity against YK-4-279 was preferentially enhanced in the ETS1 knock-down background in RKOp53KO cells (Figure 3C), suggesting critical dependence on ETS factor-mediated survival signals in this subline. This corresponds well with our finding that the expression of several other ETS factors also differed between RKOp53wt and RKOp53KO cells (compare Figure 2A, Supplementary Figure S2) and decreased upon treatment with YK-4-279 in the p53KO background (ETV1 and ETS2 in Supplementary Figure S6).

### 2.4. YK-4-279 Induces Cell Death Preferentially in a p53 Knock-Out Background 

To clarify whether hyperactivity of YK-4-279 in RKOp53KO cells is based on enhanced cytotoxicity, the time-dependency of apoptosis induction by YK-4-279 was followed by Annexin V/Propidium Iodide (PI) staining. Strikingly, gating cells according to forward scatter (FSC)/side scatter (SSC) revealed a massive appearance of debris and smaller cell fragments after 24-h exposure preferentially in the RKOp53KO cells (Figure 3D). Accordingly, profound cell disintegration became obvious from phase contrast images in this subline, while the p53wt cells displayed, in accordance with the literature, signs of mitotic cell cycle arrest [17] (Supplementary Figure S7A). Analyses of cell cycle distribution indicated pronounced YK-4-279-induced mitotic arrest (increase in G2-M phase) in both sublines (Supplementary Figure S7B). However, in the p53-deficient clone massive cell destruction, obvious as fragmented cell debris, was the dominating effect (given as the percentage of cell fragments in Supplementary Figure S7B; compare to Figure 3D). Accordingly, a moderate increase in Annexin V-positive apoptotic cells was detected in the RKOp53wt clone while in the *P53* knock-out background PI-positive but Annexin V-negative cell populations were predominantly induced (Supplementary Figure S7C). At first sight, this might suggest the induction of necrotic cell death in the RKOp53KO cells following YK-4-279 treatment. However, microscopical observations (Supplementary Figure S7A) did not support necrotic cell swelling and membrane disintegration but rather the induction of active cell fragmentation. Accordingly, Western blot analysis revealed strongly enhanced PARP1 cleavage, a classical indicator of apoptotic cell death, especially in RKOp53KO cells (Figure 3E). The pro-survival protein MCL-1 declined whereas expression of BAX, a pro-apoptotic protein overexpressed in the *P53*-deleted subline, remained unaffected. In accordance with increased PARP1 cleavage, initiator caspases 3/7 activity was significantly enhanced by YK-4-279 in the *P53* knock-out compared to the wild-type background (Figure 3F). Co-incubation with the pan-caspase inhibitor Z-VAD-FMK protected both RKO cell models moderately against YK-4-279-mediated cytotoxicity; however, the effect was more pronounced in the p53KO model (Supplementary Figure S8A). Comparable data were observed by combining YK-4-279 with the necroptosis inhibitor necrostatin (Supplementary Figure S8B). Taken together, this provides evidence that the *P53* knock-out background leads to an enhanced YK-4-279-mediated induction of a complex form of cell death including caspase-dependent and -independent components.

### 2.5. YK-4-279 Induces HyperPARylation and DNA Damage

**Figure 4:**
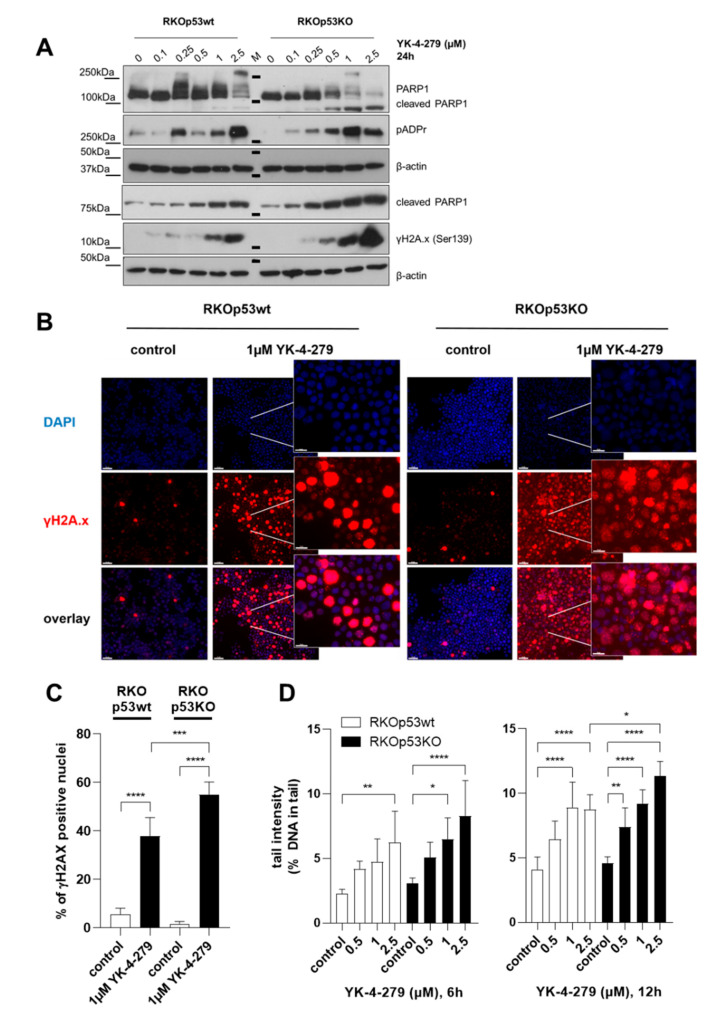
Association between the p53 status and PARylation of PARP1 as well as induction of DNA damage by YK-4-279. (**A**) Effect of YK-4-279 treatment (24 h) at the indicated concentrations on PARylation (indicated by pADPr) of PARP1 as compared to the levels of full-length PARP1, cleaved PARP1 and γH2A.x analyzed in RKOp53wt and RKOp53KO cells by Western blot. β-actin served as loading control. (**B**) Subcellular localization of γH2A.x (red) in the indicated cell models upon 24-h YK-4-279 exposure was determined by immunofluorescence staining. DAPI (blue) was used as DNA counterstain. Representative fluorescence photomicrographs are shown. The scale bar indicates 50 µm (overview) and 20 µm in the zoom-in inserts. (**C**) Quantification of the experiment under (**B**) was performed with whole-slide scanning technology (Pannoramic Digital Slide Scanner) and evaluation of at least eight regions of interest with Definiens Tissue Studio® Software. Percentage of γH2A.x-positive nuclei/mitoses upon YK-4-279 treatment in RKOp53wt as compared to RKOp53KO cells is given. Statistical analysis was conducted using unpaired Student’s *t*-test. (**D**) Induction of DNA double-strand breaks upon treatment with the indicated concentrations of YK-4-279 at the indicated time points was analyzed by Comet assay (SCGE). Data are given as mean ± SD tail intensity as published (see Material and Methods), derived from two independent experiments in triplicate. H_2_O_2_ (50 µM) served as internal positive control (not shown). Statistical analysis was performed using two-way ANOVA with Bonferroni post-test. *p* values less than 0.05 were considered significant, with * *p* < 0.05, ** p < 0.01, *** p < 0.001, **** p < 0.0001.

As the knock-down of ETS1 reduced total PARP1 (compare Figure 3A), we further investigated the impact of YK-4-279 on this base excision repair protein. Exposure to YK-4-279 dose-dependently reduced total PARP1 levels. Besides apoptotic PARP1 cleavage, hyperactivation, indicated by the addition of poly(ADP-ribose) (PAR) polymers, was also observed in response to YK-4-279 (Figure 4A). This PARylation effect, an indicator for DNA damage-induced stress response [26], was distinctly more pronounced in RKOp53KO as compared to RKOp53wt cells. Accordingly, stronger induction of DNA damage following YK-4-279 exposure was also supported by an enforced increase in γH2A.x (Ser139) in the p53KO subclone (Figure 4A–C). Unexpectedly, immunofluorescence staining revealed clear-cut differences in the localization of γH2A.x upon YK-4-279 treatment in the two subclones (Figure 4B). While in RKOp53wt cells intense staining was primarily localized at mitotic chromosomes (Figure 4B left panels), this feature was less evident in RKOp53KO cells, where a general accumulation of γH2A.x foci in the majority of interphase nuclei was observed (Figure 4B, right panels). Furthermore, a dose- and time-dependent DNA damage response upon YK-4-279 treatment was also found by a Single-Cell Gel Electrophoresis/Comet assay (SCGE) (Figure 4D). 

### 2.6. PARP Inhibition Protects RKO Cells from YK-4-279-Induced DNA Damage

**Figure 5:**
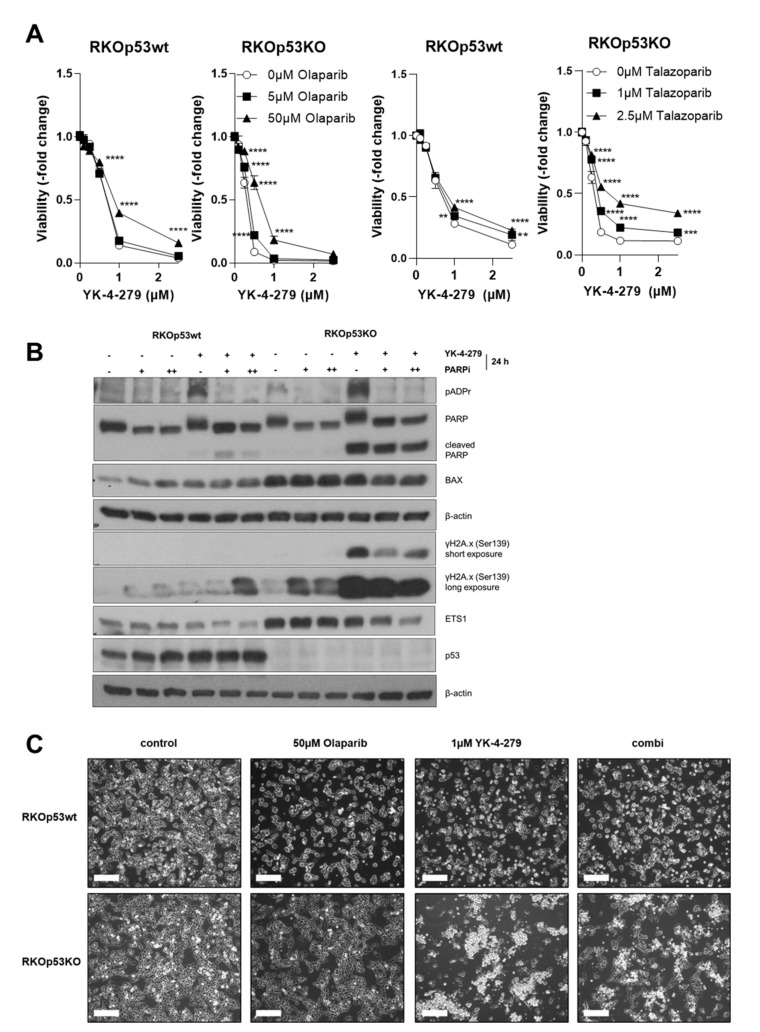
PARP inhibition protects against YK-4-279-induced cell death. (**A**) Effect of PARP inhibitors (olaparib, left panels; talazoparib, right panels) in combination with YK-4-279 for 72 h on viability of RKO subclones was determined by MTT-based cell viability assay. Results are given normalized to untreated controls as means ± SD of one representative out of three experiments performed in triplicate. Statistical analysis was conducted using two-way ANOVA with Bonferroni post-test. (**B**) Changes in PARylation of PARP1 upon 24-h YK-4-279 exposure (+, 1 µM) without or with olaparib (+, 5 µM; ++, 50 µM) are opposed to the levels of total PARP1, cleaved PARP1, BAX, γH2A.x, ETS1, and p53 by Western blot in the indicated cell models. β-actin was used as loading control. (**C**) Representative photomicrographs of RKOp53wt and RKOp53KO cells treated with the indicated drug concentrations and their combinations (combi) for 24 h. Cells were harvested and used for Western blot analysis under (**B**). The scale bar indicates 200 µm. Concerning all statistical analyses, *p* values less than 0.05 were considered significant, with ** *p* < 0.01, *** *p* < 0.001, **** *p* < 0.0001.

Considering the strong impact of YK-4-279 on PARP1 expression and especially PARylation, we tested whether PARP inhibition by the clinically approved PARP1/2 inhibitors olaparib and talazoparib impacts on the cytotoxic effect of the ETS inhibitor. Indeed, PARP inhibition significantly reduced YK-4-279 sensitivity in the RKO cell model, especially in the p53KO background (Figure 5A). PARP inhibitors efficiently blocked PARP1 PARylation and induced moderate levels of H2A.x phosphorylation, especially in the p53KO subline. In the combination setting, PARP inhibition reduced YK-4-279-induced PARP1 cleavage and H2A.x phosphorylation selectively in the RKOp53KO subline (Figure 5B). This is well in accordance with the antagonistic effect observed in the cell viability assay (Figure 5A). However, inhibition of ETS1 expression by YK-4-279 tended to be enforced by a blockade of PARP1 (Figure 5B). This suggests a role of PARP1 enzyme activity in the regulation of ETS1 signaling, especially in a *P53* knock-out cellular background. Representative photomicrographs showing a clear rescue effect of the drug combination in the *P53* knock-out subline are depicted in Figure 5C. A pronounced antagonistic activity of PARP inhibitors in combination with YK-4-279 was also observed in several melanoma models preferentially in p53 non-responsive cases (Supplementary Figure S9).

### 2.7. YK-4-279 Induces PARP1-Dependent Cell Death 

**Figure 6:**
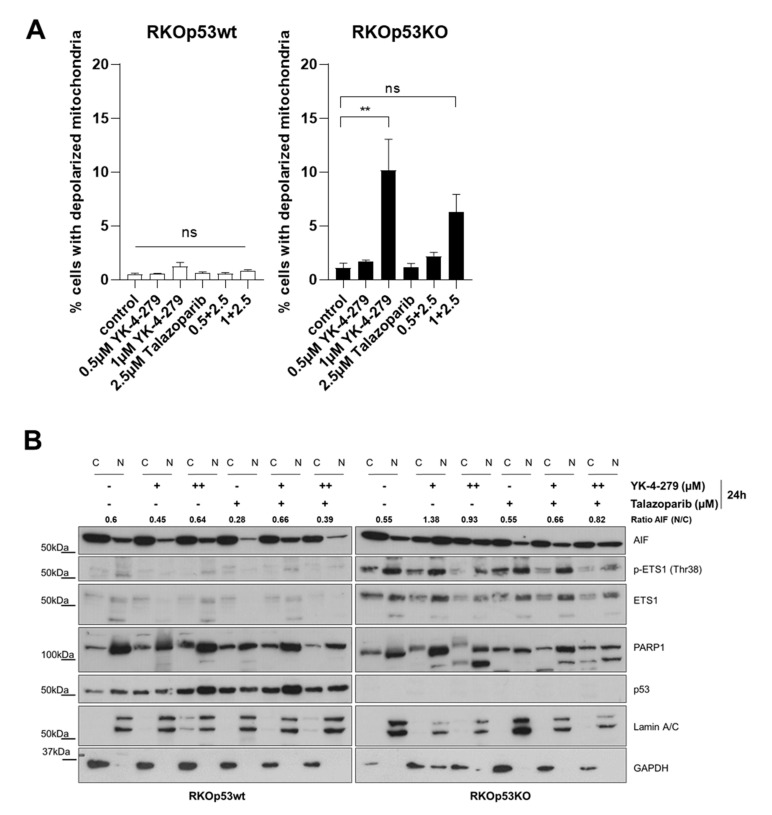
YK-4-279 induces parthanatos-like cell death. (**A**) Impact of YK-4-279 as indicated in absence or presence of talazoparib for 24 h on the mitochondrial membrane potential of RKOp53wt and RKOp53KO cells were analyzed with JC-1 staining followed by flow cytometry. Data are given as means ± standard error of mean (SEM) of three independent experiments. One-way ANOVA with Bonferroni post-test was performed to test for statistical significances. *p* values < 0.05 were considered significant, with ** *p* < 0.01. ns, not significant. (**B**) Changes in the amounts and subcellular localization of apoptosis-inducible factor (AIF), p-ETS1, ETS1, PARP1 and p53 were analyzed in nuclear (N) and cytoplasmic (C) fractions of RKOp53wt and RKOp53KO cells after exposure to YK-4-279 (+, 0.5 µM; ++, 1 µM) without or with talazoparib (+, 2.5 µM) for 24 h. Lamin A/C and GAPDH were used as purity control for nuclear extracts and cytoplasmic fractions, respectively. Densitometric quantification of nuclear versus cytoplasmic fractions of AIF is given as ratio of AIF (N/C) insets.

Both the profound YK-4-279-induced hyperPARylation and the antagonistic effects of PARP inhibitors suggest a central role of PARP activity in the YK-4-279-mediated cell death induction, especially in a p53-deficient background. These features characterize a special form of PARP1-dependent programmed cell death designated parthanatos. The hallmarks of parthanatos are activation and poly(ADP)ribosylation of PARP1, translocation of the apoptosis-inducible factor (AIF) from mitochondria to nuclei as well as DNA fragmentation [26,27,28,29]. Additionally, mitochondrial membrane depolarization and independency of caspase activity for programmed cell death induction can contribute to this specific cell death phenotype [30]. Accordingly, YK-4-279 triggered a dose-dependent increase in PARylation of PARP1 in RKOp53wt and RKOp53KO cells (see Figure 4A). Additionally, depolarization of mitochondrial membranes of YK-4-279-treated RKOp53KO cells was partially inhibited by addition of the PARP inhibitor talazoparib (Figure 6A). YK-4-279 induced the translocation of AIF from mitochondria to nuclei in the RKOp53KO cells already at 0.5µM YK-4-279, one of the most specific features of parthanatos [28]. This translocation was again inhibited by co-exposure to talazoparib (Figure 6B). 

## 3. Discussion

Know-how concerning molecular factors regulating the anti-cancer activity of YK-4-279, originally designed to target oncogenic ETS factor fusion protein interactions in ES, has only started to emerge recently [17,31]. At the molecular level, YK-4-279 blocks the interaction of an oncogenic EWS/ETS fusion protein with its cofactor RHA. However, YK-4-279 does not only exert anti-cancer activity in ES [8,10,11,24], but also in prostate cancer [12,13,32,33], lymphoma [14], neuroblastoma [17,31] and glioma [10,15]. Ongoing research uncovered that YK-4-279 can also interfere with the mitotic machinery, leading to a pronounced mitotic arrest, and displays synergistic effects with vincristine [34]. Although the specific mechanisms underlying mitotic arrest induction upon YK-4-279 treatment are still under debate; they may include EWS/FLI1-activated UBEC2 to control cyclin B1 levels [34] as well as ETS factor-independent activities—e.g., via aurora kinase interactions [17]. Here, we demonstrate that YK-4-279 in vitro anti-cancer efficacy is distinctly enhanced by *P53* deletion in an ETS fusion-negative, but BRAF^V600E^-positive, colon cancer background. Molecularly, the sensitizing effect of p53 loss/deregulation was based on ETS1 overexpression and consequent YK-4-279-mediated parthanatos induction.

An in vitro human cell model screen for YK-4-279 responsiveness revealed a surprising hyperactivity of this compound in the *P53* knock-out subline of the human colon carcinoma cell model RKO, resulting in an IC_50_ value in the lower nanomolar range. Enhanced activity of YK-4-279 associated with p53 deregulation was in this study also observed in a melanoma and ES background. In case of melanoma, p53 mutations are comparably rare; however, p53-mediated signal transduction is often impeded even in p53 wild-type tumors [35]. Consequently, we focused here on responsive versus non-responsive melanoma models in terms of p53-mediated p21 activation following DNA damage, as published previously [35]. Additionally, ES cells are widely p53 wild-type, but effective p53 signaling needs to be suppressed during Ewing´s sarcomagenesis either directly by EWS/FLI1 itself and/or by deregulation of p53 upstream effectors including MDM2/MDM4 [36,37]. 

Little is known on the impact of the p53 status on sensitivity against ETS factor inhibition by YK-4-279. Kollareddy et al. stated that the ETS translocation-independent, anti-neuroblastoma activity of YK-4-279 was not reliant on wild-type p53 signaling [17]. Concerning ES, van der Ent et al. [24] solely demonstrated that the ETS inhibitor activates p53 expression in p53wt ES cells but concluded that EWS/FLI1 inhibition-mediated cell death is independent of p53 signaling. Nevertheless, in this study the *P53*-deleted ES cells were most sensitive against YK-4-279 monotherapy, thus resembling our data. Additionally, our study revealed a massive induction of p53 by YK-4-279 in the p53 wild-type RKO cell model. Van der Ent et al. suggest that in the EWS/FLI1-driven ES background this p53 induction underlies the synergistic effect of YK-4-279 with the p53 stabilizer Nutlin-3. While confirming this synergism in a p53 wild-type, EWS/FLI1-driven ES cell model (FP-BH), we found a strong antagonistic effect of Nutlin-3 and YK-4-279 especially in p53/p21-responsive melanoma models. This suggests, that in an ETS translocation-negative background, YK-4-279 targets an ETS factor differing critically from EWS/FLI1 in its interaction with wild-type p53. Moreover, at least in the BRAF-mutated cancer background, intact p53 signaling might rather protect than sensitize against YK-4-279-mediated cell death initiation. 

In search for factors underlying the YK-4-279-protective effects of wild-type p53, we screened expression changes with a focus on potential YK-4-279 targets, namely ETS factors. These transcription factors represent critical mediators for RAS/MEK/ERK signaling [5,38] and activated ETS molecules that are able to mimic MAPK hyperactivation in prostate cancer [2]. Accordingly, ETS factor inhibition has even been suggested as a potential substitute for upstream MAPK blockades at the level of MEK in MAPK-hyperactivated tumors [15,22]. Loss of p53 in RKO cells induced distinct overexpression of *ETS1*, *ELK3*, *ELF1*, and *SPIB* at the mRNA level. In our previous study concerning BRAF^V600E^-positive high-grade glioma, ETS1 was the only ETS factor affected by BRAF inhibition [15]. Taking into account the BRAF mutation status of the RKO model, we decided to focus on this critical oncogenic ETS factor [39] for further analyses. Indeed, *P53* deletion induced massive ETS1 overexpression in the BRAF-mutant RKO cell model, while this effect was widely missing in the BRAF wild-type HCT116 cells. Accordingly, YK-4-279 hypersensitivity following *P53* deletion was comparably mild in the HCT116 model, again pointing toward a central role of ETS1 in the BRAF-mutated RKO cells. In line with this hypothesis, ETS1 overexpression in RKOp53KO cells was completely reversed by an upstream BRAF blockade using dabrafenib. Correspondingly, RKOp53KO cells displayed a clear dabrafenib hypersensitivity, suggesting an enhanced oncogenic potential of ETS1, and in turn YK-4-279 sensitivity, as a consequence of *P53* deletion.

So, the question remains as to how wild-type p53 restrains MAPK-induced ETS1 expression in the BRAF-mutated RKO cell background. In accordance with our observation on increased *ETS1* mRNA levels, especially in the RKOp53KO cell model, Iotsova et al. reported that wild-type p53 is able to directly repress the TATA-less *ETS1* gene promoter [40]. Additionally, at the protein level, ETS1 and ETS2 have been shown to interact with wild-type and mutant p53 with different affinities [18,19,20,21]. The ETS1/wild-type p53 interaction especially induced efficient ETS1 degradation, and p53 knock-out caused profoundly enhanced ETS1 expression levels in mouse embryonic fibroblasts (MEFs) [21]. Together, these data suggest that YK-4-279 might reverse the well-described cell death resistance mediated by the loss of wild-type p53 [41], partly based on interactions with oncogenic ETS1 signals especially in an MAPK-driven background. 

Concerning the mechanisms underlying YK-4-279-induced cytotoxicity, we found several features of DNA damage induction. Hence, marked H2A.x phosphorylation was detected already at distinctly lower YK-4-279 concentrations in the RKOp53KO as compared to the RKOp53wt model. Surprisingly, the pattern of γH2A.x immunofluorescence staining in response to YK-4-279 markedly differed according to p53 status. γH2A.x almost constitutively accumulated at metaphase chromosomes of YK-4-279 mitosis-arrested RKOp53wt but only rarely on those of RKOp53KO cells. On the contrary, YK-4-279 also induced massive accumulation of classical γH2A.x foci in interphase nuclei in the p53KO model. Induction of DNA double-strand breaks by YK-4-279 was also proven by SCGE assay; however, the difference according to p53 status was less pronounced as compared to γH2A.x staining. This implicates that not the entire YK-4-279-induced γH2A.x response is a consequence of double-strand break induction. Accordingly, DNA damage-independent γH2A.x accumulation at metaphase chromosomes has been observed in several proliferating cancer cell types in a p53-independent fashion [42]. In contrast, the YK-4-279-induced hyperaccumulation of γH2A.x specifically at metaphase chromosomes, as observed in our study, was predominantly found under wild-type p53 conditions. The specific mechanisms underlying mitotic arrest induction upon YK-4-279 treatment are still under debate and include EWS/FLI1-activated UBEC2 to control cyclin B1 levels [34] as well as aurora kinase interactions [17]. Whether these mechanisms also contribute to our observation concerning γH2A.x activation needs to be worked out in detail. 

Proper recruitment of γH2A.x and other repair proteins including p53 at damaged DNA sites is supported by the DNA double-strand sensor PARP1 [25]. Accordingly, YK-4-279 induced the hyperPARylation of PARP1 at lower concentrations in the absence of p53, thus resembling γH2A.x induction. These data implicate that the otherwise protective DNA damage response is linked to increased cell death induction upon ETS factor inhibition by YK-4-279. Accordingly, YK-4-279-induced cell death also revealed, besides hyperPARylation, other cellular processes, including mitochondrial membrane depolarization and nuclear AIF translocation, characteristic of a PARP1-regulated form of cell death, designated parthanatos [43]. This specific form of cell death has not been associated with ETS factor inhibition so far, and a regulatory role of p53 is currently enigmatic. However, parthanatos-related cell death was previously found in BRAF^V600E^-mutated, MAPK-inhibitor-resistant and -dependent human melanoma cells upon drug withdrawal (drug holiday) [44]. In our study, parthanatos-like cell death features were predominantly found in the p53KO model while widely lacking in RKOp53wt cells. Moreover, pharmacological PARP inhibition with olaparib or talazoparib impeded YK-4-279-induced cell death significantly stronger in the *P53*-deleted subline. Together, this points toward a parthanatos-inhibiting function of ETS proteins and especially ETS1, preferentially in a BRAF^V600E^-mutated background after loss of p53. Indeed, PARP1 overexpression induced by *P53* deletion in RKOp53KO cells was distinctly reduced by *ETS1* mRNA knock-down. This is in line with a previous report demonstrating that ETS1 is the predominant ETS factor binding and activating the *PARP1* gene promoter even in a EWS/FLI1 fusion-positive ES background [45]. The antagonism of PARP inhibitors with YK-4-279 activity was confirmed in a p53/p21 non-responsive melanoma context. Consequently, our study identified a novel mode of hyperPARylation-mediated cell death induction by the ETS factor inhibitor YK-4-279. Therefore, ETS factor inhibitors should represent a promising therapeutic option for chronically therapy-resistant tumors with p53 signaling defects especially in conjunction with activating MAPK pathway alterations. 

## 4. Materials and Methods 

### 4.1. Reagents 

YK-4-279 (S7679), Nutlin-3 (S1061), talazoparib (S7048), necrostatin (S8037), Z-VAD-FMK (S7023), and dabrafenib (S2807) were purchased from Selleck Chem (Houston, TX, USA), olaparib (O-9201) from LC Laboratories (Woburn, MA, USA), cisplatin and a 30% H_2_O_2_ solution was purchased from Sigma Aldrich (St Louis, MO, USA). All compounds, except cisplatin and H_2_O_2,_ were dissolved in dimethylsulfoxid (DMSO; Sigma Aldrich) obtaining a final stock concentration of 10 mM. For cisplatin a 5 mM stock in cell culture medium was made. These compounds were used in a range from 0–50 µM. 

### 4.2. Cell Models 

RKOp53wt colon cancer cells together with the RKOp53KO (homozygous knock-out) subline as well as HCT116p53wt colon cancer cells and the HCT116p53KO subline were generously provided by Dr. Vogelstein from John Hopkins University, Baltimore. All colon cancer cell models and their respective sublines were cultured in McCoy’s medium (Sigma Aldrich) supplemented with 10% fetal calf serum (FCS; PAA, Linz, Austria) and 2-mM glutamine (Sigma Aldrich). FP-BH was established from a surgical specimen obtained from the Orthopaedic Clinic at Hospital Lainz, (Vienna, Austria) at the Institute of Cancer Research, Medical University of Vienna, Austria and cultured in Roswell Park Memorial Institute medium (RPMI)-1640 growth media (Sigma Aldrich) containing 10% FCS. RD-ES (HTB166) cells were obtained from American Type Culture Collection (ATCC; Manassas, VA, USA) and cultured in Iscove’s Modified Dulbecco’s medium (IMDM, Thermo Fisher Scientific, Waltham, MA, USA) containing 10% FCS. VM48, VM1, VM47 and VM18 cell models were established from surgical specimens at the Institute of Cancer Research, Medical University of Vienna, Austria. Details on the origin of the melanoma models are given in Pirker et al. [35]. Cells were cultured with RPMI-1640 growth media containing 10% FCS. All cell lines were cultured without addition of any antibiotics at 37 °C and 5% CO_2_. Cells were routinely tested for *Mycoplasma* infections. Short tandem repeat (STR) profiling was performed for cell line authentication.

### 4.3. Cell Viability Assay (MTT)

According to their proliferation rate. 2–4 × 10^4^ cells/mL were plated in 100 µL medium into 96-well plates and were allowed to settle for 24h. On the following day, drugs (YK-4-279 without or with olaparib, talazoparib, Nutlin-3, dabrafenib, necrostatin or Z-VAD-FMK) were added. After 72 h of drug exposure, cell viability was determined by 3-(4,5-dimethylthiazol-2-yl)-2,5-diphenyltetrazolium (MTT)-based vitality assay (EZ4U kit, Biomedica, Vienna, Austria) as published [46,47]. Cytotoxicity of YK-4-279 was expressed using half-maximal inhibitory concentration (IC_50_) values calculated from full dose-response curves. Raw data were analyzed with GraphPad Prism 8.0.1 (GraphPad Software, San Diego, CA, USA) and the final results were normalized to either the solvent control (single setting) or the respective single drug treatment without YK-4-279 (combination setting). Data are given as mean ± standard deviation (SD). 

### 4.4. Colony Formation Assay 

In total, 1 × 10^3^ cells/well were seeded into 24-well plates. On the following day, drugs were added. After drug incubation for seven days, cells were fixed with ice-cold methanol for 20 min at 4°C and stained with crystal violet. For quantification, plates were scanned on a Typhoon TR0 Variable Mode Manager (GE Healthcare Bioscience, Uppsala, Sweden) using the software Typhoon Scanner Control v5.0. Analysis of raw data was performed with Fiji (Version 1.52p) and GraphPad Prism 8.0.1. Data were normalized to the untreated control and are given as mean ± SD. 

### 4.5. Whole Genome Gene Expression Arrays

mRNA of RKOp53wt and RKOp53KO cells was isolated with an RNeasy Mini Kit from Qiagen (Hilden, Germany) according to the manufacturer’s instructions. Quality and integrity of mRNA was checked on the Agilent 2100 Bioanalyzer, and only samples with RIN values >9 were included for microarray analysis. Whole genome oligonucleotide-based gene expression microarray analysis was performed using the 4 × 44 K microarray format as described previously [48]. Dual-color experiments were performed using the Quick Amp Labelling Kit according to the instructions provided (Agilent, Santa Clara, CA, USA). Slides were scanned on a G2505B Micro Array Scanner (Agilent). Feature extraction and data analysis were carried out using the Feature Extraction and GeneSpring software (both Agilent).

### 4.6. Flow Cytometry-Based Determination of Cell Death Induction, Mitochondrial Depolarization, and Cell Cycle Progression 

For cell death and cell cycle analyses, 4 × 10^5^ cells/ml were plated in 6-well plates containing 2 mL of growth medium and cells were allowed to settle for 24 h before addition of increasing concentrations of YK-4-279. For Annexin V-based cell death analysis, performed as described previously [49], cells were treated for 24 h or 48 h and then stained with fluorescein isothiocyanate (FITC) or allophycocyanin (APC)-labelled Annexin V (BD Bioscience, Franklin Lakes, NJ, USA) and propidium iodide (PI) (Sigma Aldrich) before flow cytometric analysis using BD Calibur (BD Bioscience, Franklin Lakes, NJ, USA). Mitochondrial membrane depolarization was analyzed by JC-1 staining as published [50]. Cells were treated with increasing concentrations of YK-4-279 (0.5 µM, 1 µM) without or with talazoparib (2.5 µM). After 24 h, drug incubation cells were harvested and stained with 10-µg/mL JC-1 iodide (Enzo Life Sciences, Inc., New York, NY, USA) for 20 min. For cell cycle analyses, cells were treated for 24 h with YK-4-279, followed by propidium iodide (PI) staining and analysis by flow cytometry, as published [35]. Annexin V staining and cell cycle analyses were quantified using the CellQuest Pro software (BD Bioscience), and data were illustrated using GraphPad Prism 8.0.1. In the case of JC-1 staining, samples were immediately subjected to flow cytometric analysis on a BD Fortessa, and data were analyzed using FlowJo v10.06 and illustrated using GraphPad Prism 8.0.1.

### 4.7. Total Protein Isolation and Western Blot

Briefly, 3–4 × 10^5^ cells/mL were plated in 6-well plates and, after 24 h, treated with the indicated concentrations of YK-4-279 without or with olaparib or Nutlin-3 for 24 h. Cell were scratched, washed with PBS and chemically (buffer containing: 50 mM Tris/HCl (pH 7.6), 300 mM NaCl, 0.5% Triton X-100, protease inhibitors (phenylmethylsulfonyl fluoride (PMSF), complete and phosphatase inhibitor PhosSTOP; all supplements from Roche, Rotkreuz, Switzerland) and mechanically (ultrasound) lysed. Determination of the total protein concentration was performed according to the manufacturer’s instruction (Pierce™ BCA Protein Assay Kit, Rockford, IL, USA). In total, 15µg of total protein extracts were separated by sodium dodecyl sulphate–polyacrylamide gel electrophoresis (SDS-PAGE). Subsequently, proteins were blotted onto polyvinylidene difluoride membranes (PVDF, Thermo Fisher Scientific) and Ponceau protein staining was performed to confirm blotting efficacy. A detailed list of the antibodies used is given in the Table S1. 

### 4.8. Nuclear and Cytoplasmic Protein Isolation

In total, 1.5 × 10^6^ cells were plated into 60-mm dishes. After 24h, cells were treated with the indicated concentrations of YK-4-279 without or with talazoparib. Isolation was performed using the NE-PER Nuclear and Cytoplasmic Extraction Kit (Thermo Scientific) according to manufacturer’s instructions. Determination of the protein concentration as well as Western blot analysis was performed as described above.

### 4.9. Immunofluorescence Staining

Briefly, 3 × 10^5^ cells/ml were seeded in 300 µL into an 8-chamber coverslip with removable silicone walls (Ibidi GmbH, Gräfeling, Germany). Next, cells were fixed with 4% paraformaldehyde (PFA) for 15 min at RT, washed with PBS and a PBS-based blocking solution containing 5% FCS. 0.3% Triton-X-100 was added for 1 h at RT. Cells were again washed with PBS and the primary ETS1 antibody (Cell Signaling, #14069, 1:3200) was added for overnight incubation at 4 °C. After removal of the primary antibody, three washing steps with PBS and an incubation with secondary antibody (Goat anti-Rabbit IgG (H + L) Cross-Adsorbed Secondary Antibody, Alexa Fluor 594, A11029, Thermo Fisher Scientific) for 1 h at RT was performed. After three washing steps with PBS, cells were counterstained with 4′,6-diamidino-2-phenylindole (DAPI, Thermo Fisher Scientific). Analysis was performed on a Zeiss LSM700 confocal microscope (Carl Zeiss Microscopy GmbH, Jena, Germany) using a 63x oil immersion objective. In the case of γH2A.x staining, 4 × 10^5^ cells/mL were plated in 6-well plates and after 24 h cells were treated with the indicated concentrations of YK-4-279. Following 24 h of drug incubation and a washing step, cells were centrifuged onto Super Frost slides (400 rpm for 5 min) using a Cytospin™ 4 Cytocentrifuge (Thermo Fisher Scientific). Cells were fixed with acetone:methanol (1:1) for 10min at -20°C. Staining of γH2A.x was performed as described above using a 1:200 dilution. Scanning of slides was performed using a Pannoramic Digital Slide Scanner, and analysis was performed using Definiens Tissue Studio® Software (subsidiary of AstraZeneca, Cambridge, UK) and GraphPad Prism 8.0.1. Contrast and brightness were adjusted with Fiji/Image J (Version 1.52p).

### 4.10. RNA Isolation and qRT-PCR

Briefly, 3 × 10^5^ cells/ml were seeded into 6-well plates. Total RNA isolation was conducted using TRIzol (Life technologies, Thermo Fisher Scientific) reagent and chloroform according to standard protocols. RNA concentration and purity (ratio 260/280) were assessed on a Nanodrop 1000 (Thermo Fisher Scientific). In total, 500 ng of RNA was reverse transcribed into cDNA with Revert Aid Reverse Transcriptase (Thermo Fisher Scientific). For qRT-PCR, cDNA was diluted 1:25 and mixed with 2x GoTaq Green Master Mix (Promega, Madison, WI, USA) and 10nM of specific forward and reverse primers (Eurofins Scientific, Luxembourg, Luxembourg; primer sequences in Supplementary Table S2). qRT-PCR was performed and analyzed on a CFX Connect Real-Time PCR Detection System. For analysis, data were normalized to the internal control gene *RPL-41* for calculation of ∆Ct values.

### 4.11. Gene Knockdown by siRNA

RKOp53wt and RKOp53KO cells were transfected with siRNA against *ETS1* (Accell Human ETS1 siRNA SMARTpool, E-003887-00-0005, Dharmacon, Lafayette, CO, USA) or non-targeting (scrambled, scr) siRNA (Accell Green Non-targeting siRNA, D-001950-01-05, Dharmacon, Lafayette, CO, USA) following the manufacturer’s recommendation. Briefly, for cell viability analysis, 1.5 × 10^4^ cells/mL were seeded, incubated for 72 h with the siRNA, followed by another 72-h incubation with increasing YK-4-279 concentrations. A cell viability (MTT) assay was performed as described above. For Western blot analysis for proving the efficacy of the ETS1 knock-down, 2 × 10^5^ cells/ml were seeded, exposed to the siRNA for 72 h and processed for protein isolation and Western blot as described. 

### 4.12. Single-Cell Gel Electrophoresis/Comet Assay (SCGE)

Briefly, 4 × 10^5^ cells/mL were seeded into 6-well plates and treated on the following day with YK-4-279 (0.5, 1, 2.5 µM) for 6 h or 12 h. After treatment, cells were harvested and 3 × 10^5^ cells/condition were re-suspended in 300 µL cell culture medium. Only cultures with a viability ≥70% were evaluated in SCGE assays [50,51]. In total, 50 µL of the cell suspension was mixed with low melting point agarose (300 µL, 0.5% LMPA), spread onto pre-coated agarose slides (1.5% NMPA) and lysed in the dark at 4 °C for 1 h. After unwinding for 25min under alkaline conditions (pH >13), electrophoresis (300 mA, 1.0 V/cm at 4 °C for 25 min) was performed. Neutralization with PBS was carried out twice for 8min. Slides were stained with propidium iodide (10 µg/mL) and air-dried. The percentage of DNA in the comet tails was analyzed by an automated image analysis system (Comet IV, Perceptive Instruments Ltd., Burry St. Edmunds’, UK). Two independent experiments were performed (3 slides/concentration and experiment; n=6/concentrations) and 50 nuclei/slide were randomly selected for evaluation.

### 4.13. Caspase-3/7 Activity

Briefly, 4 × 10^4^ cells/mL were plated into white, flat-bottom 96-well plates (100 µL/well). On the following day, cells were treated with the indicated concentrations of YK-4-279 or cisplatin. After 24 h of drug incubation, caspase activity was determined by using the Caspase-Glo® 3/7-assay (G8091) according to the manufacturer’s instructions (Promega). 

### 4.14. Statistical Analysis

Data are expressed as mean ± standard deviation (SD) or standard error of mean (SEM) as indicated in the respective figure legends. Results were analyzed and illustrated using GraphPad Prism 8.0.1 Software and Fiji/Image J (Version 1.52p). All experiments, if not otherwise stated, were performed at least three times. Statistical analyses and significances are described in the respective figure legends and indicated by asterisks in the figures (* *p* < 0.05, ** *p* < 0.01, *** *p* < 0.001, and **** *p* < 0.0001).

## 5. Conclusions

In conclusion, we have uncovered that loss of p53 promotes the sensitivity of cancer cells against the ETS factor inhibitor YK-4-279, especially in a MAPK-driven oncogenic background. We further identified induction of a parthanatos-like form of cell death by YK-4-279 based on a deregulated MAPK/ETS1/p53/PARP1 signaling axis. Consequently, these data suggest a novel and biomarker-driven therapeutic strategy for p53-deleted tumors, generally characterized by high intrinsic therapy resistance.

## Supplementary Materials

The following are available online at https://www.mdpi.com/2072-6694/12/11/3205/s1, Figure S1: Cancer cells with p53 loss-of-function are more susceptible to YK-4-279 treatment, Figure S2: ETS factor mRNA expression in RKOp53KO as compared to RKOp53wt cells, Figure S3: BRAF inhibition protects against YK-4-279-mediated cytotoxicity, Figure S4: Stabilization of wild-type p53 expression by Nutlin-3 reduces ETS1 protein levels, Figure S5: Nutlin-3 protects against the cytotoxic effect of YK-4-279 in p53/p21-responsive melanomas, Figure S6: ETV1 and ETS2 protein levels are affected by YK-4-279 in the RKOp53KO model, Figure S7: YK-4-279-induced cell death and cell cycle arrest in relation to p53 status of RKO cells, Figure S8: Minor protection by the pan-caspase inhibitor Z-VAD-FMK and the necroptosis inhibitor necrostatin against YK-4-279 cytotoxicity, Figure S9: PARP inhibition protects melanoma cells against YK-4-279-induced cytotoxicity, Table S1: Antibodies, Table S2: Primers for qRT-PCR.
