# Supplementary material for: p53 Loss Mediates Hypersensitivity to ETS Transcription Factor Inhibition Based on PARylation-Mediated Cell Death Induction
Source: Cancers (Basel). 2020 Oct 30;12(11):3205. doi: 10.3390/cancers12113205 (PMC7693367; doi:10.3390/cancers12113205)
Supplement: Supplementary file 1 [file cancers-12-03205-s001.zip › cancers-949334-sup/Figures S1-S9_Dinhof et al.pdf]

Figure S1 Dinhof et al.

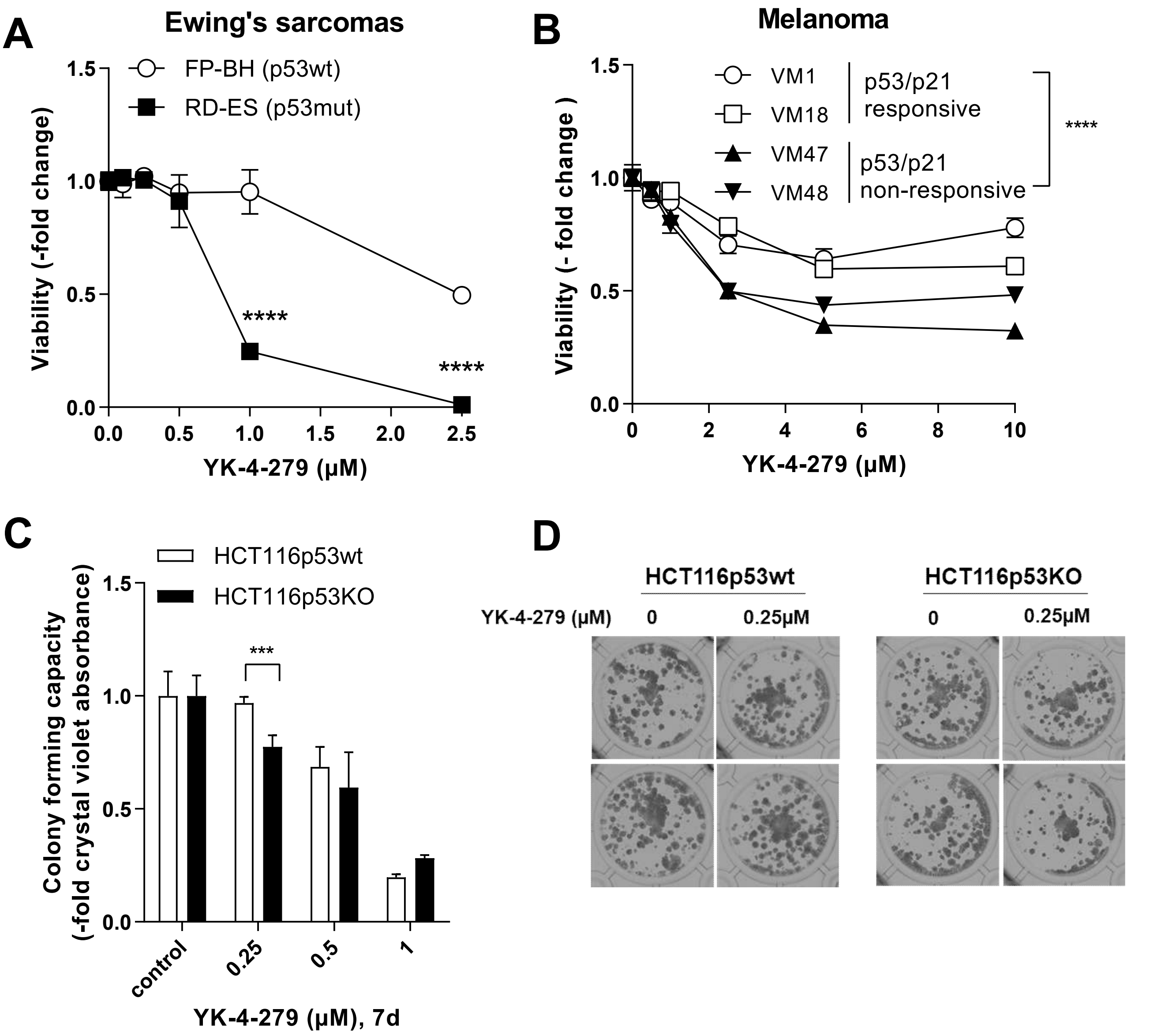

Figure S1: Cancer cells with p53 loss-of-function are more susceptible to YK-4-279 treatment. (**A, B**) Impact of a 72h exposure to YK-4-279 at the indicated concentrations on viability of (**A**) Ewing`s Sarcoma cells (FP-BH, p53wt; RD-ES, p53mut) and (**B**) melanoma cells (VM1, VM18, p21/p53 responsive; VM47, VM48, p21/p53 non-responsive) was determined by MTT-based cell viability assay. Values are given as mean ± SD of one representative out of three experiments performed in triplicate. (**C**) Effect of YK-4-279 on clone forming capacity of the colon carcinoma cell models HCT116p53wt and HCT116p53KO after 7 days of incubation with the indicated concentrations of YK-4-279 was determined by colony formation assay followed by crystal violet staining and fluorescence intensity measurement on Typhoon TR0 Variable Mode Manager. Values shown are given normalized to untreated controls as means ± SD of one representative out of three experiments performed in triplicate. (**D**) Representative photographs for (**C**) are shown. Two-way ANOVA with Bonferroni post-test was used for statistical testing. p values less than 0.05 were considered significant, with *** $p < 0.001$, **** $p < 0.0001$.

Figure S2

Dinhof et al.

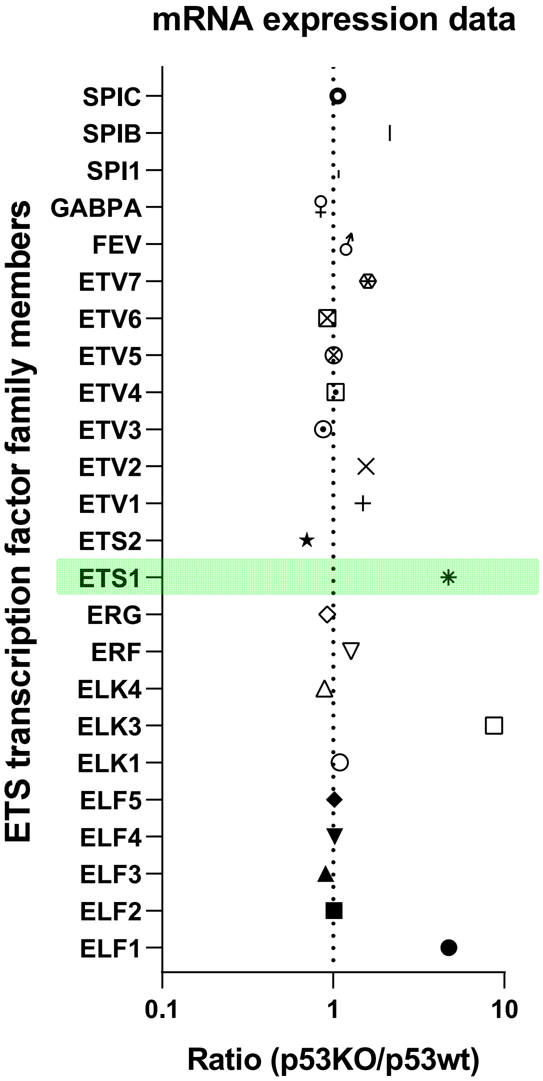

Figure S2: ETS factor mRNA expression in RKOp53KO as compared to RKOp53wt cells. Whole genome mRNA expression was determined using 44K whole genome gene expression microarrays and the GeneSpring software (Agilent). Differential mRNA expression of 24 ETS transcription factor family members is given as ratio between RKOp53KO and RKOp53wt. ETS1 is highlighted in green.

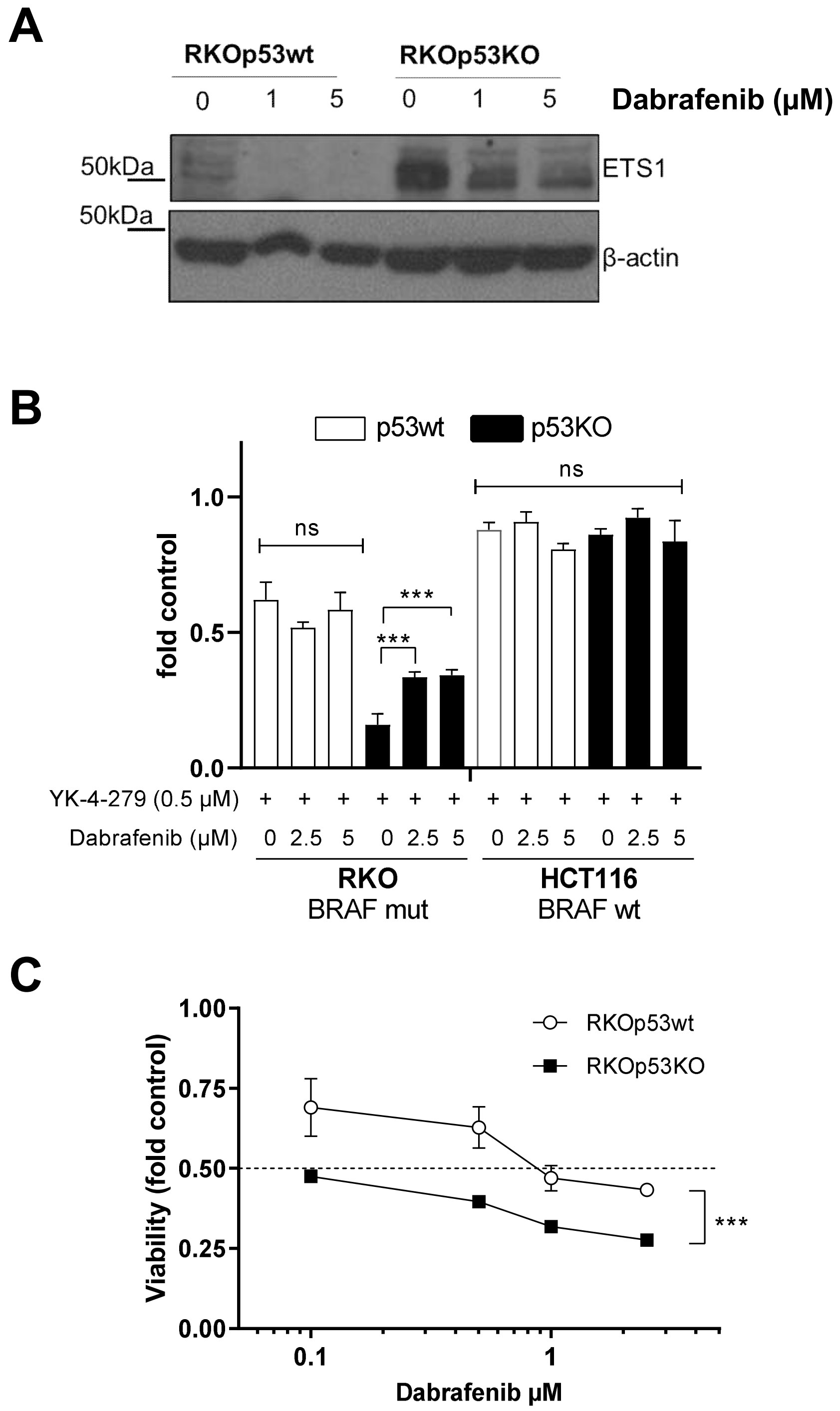

Figure S3: BRAF inhibition protects against YK-4-279-mediated cytotoxicity. (**A**) Impact of a 24h dabrafenib treatment at the indicated concentrations on the expression of ETS1 in the RKO cell model. (**B**) Impact of 72h dabrafenib treatment in combination with YK-4-279 at the indicated concentrations on cell viability of the $BRAF^{V600E}$-mutant RKOp53wt and RKOp53KO as well as of the BRAF wild-type HCT116p53wt and HCT116p53KO cells was determined by MTT-based cell viability assay. Statistical significance was tested by Student's t test. (**C**) Activity of dabrafenib for 72h as single agent in the RKO colon cancer cell model as indicated. Values are given as mean ± SD of one representative out of three experiments performed in triplicate. Statistical analysis was done using two-way ANOVA. p values less than 0.05 were considered significant, with *** p < 0.001.

Figure S4

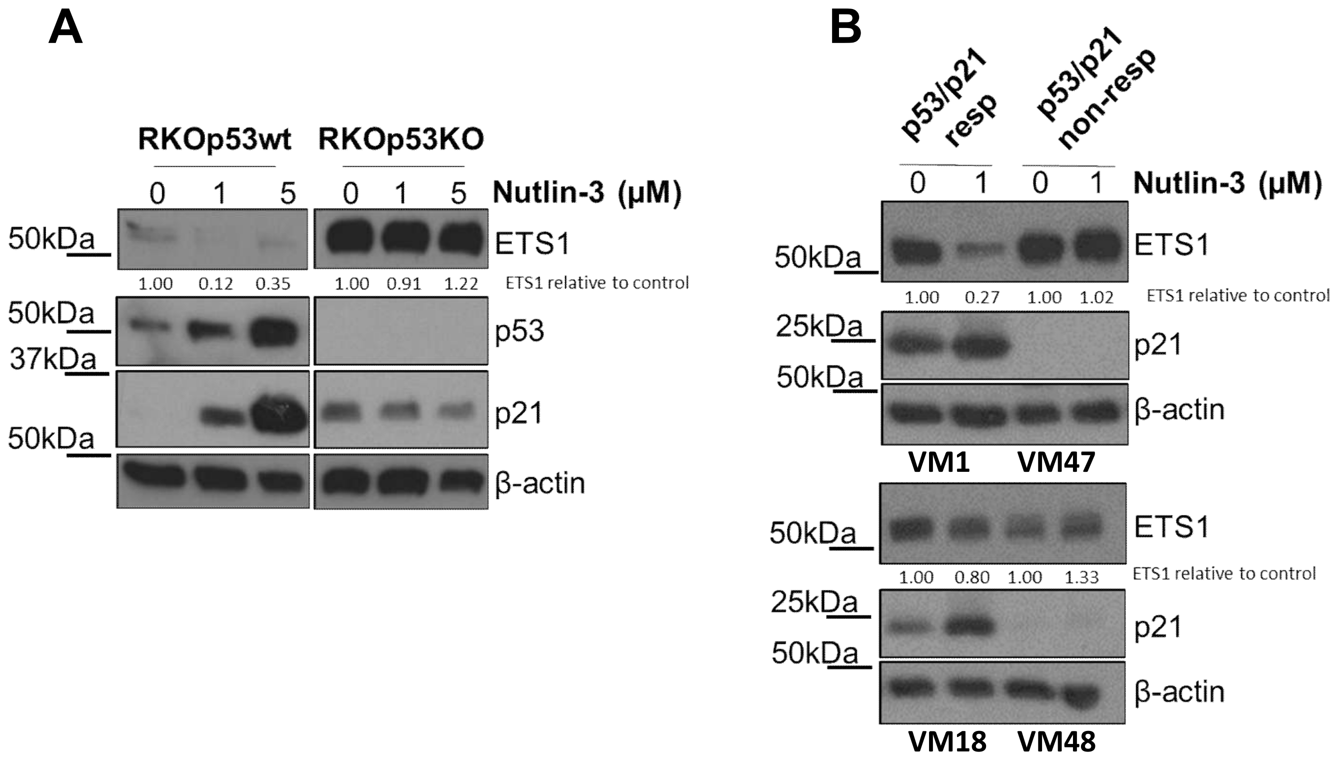

Figure S4: Stabilisation of wild-type p53 expression by Nutlin-3 reduces ETS1 protein levels. Effects of Nutlin-3 treatment at the indicated concentrations for 24h on the expression of ETS1 in (**A**) the RKO cell model as well as (**B**) p53/p21-responsive (VM1, VM18) and non-responsive (VM47, VM48) melanoma models determined by Western blot are depicted. Data were quantified by densitometric analyses (number insets below the ETS1 bands) relative to β-actin used as loading control.

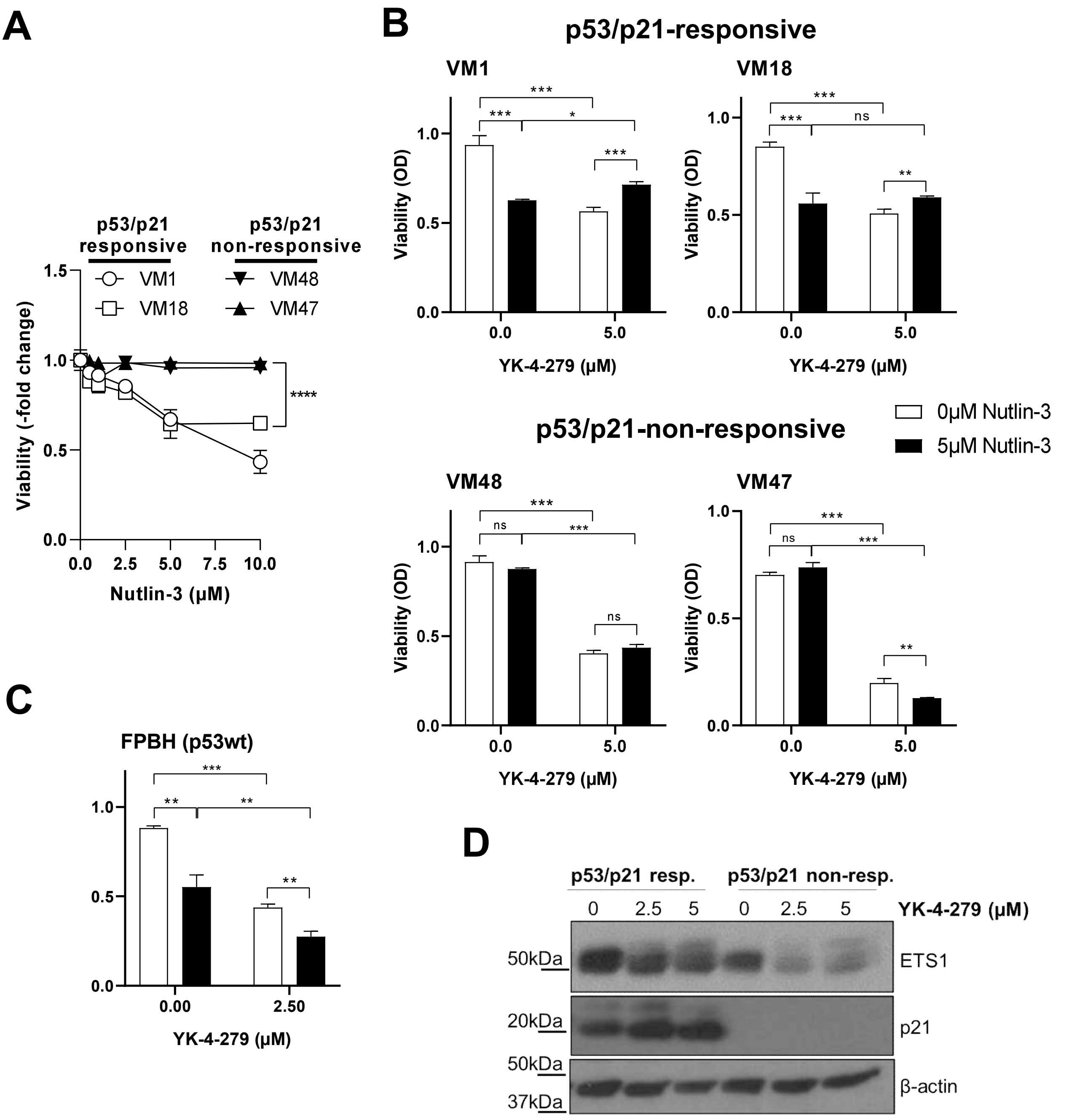

Figure S5: Nutlin-3 protects against the cytotoxic effect of YK-4-279 in p53/p21-responsive melanomas. (**A)** Effect of Nutlin-3 treatment for 72h at the indicated concentrations on the viability of p53/p21-responsive (VM1, VM18), and -non-responsive (VM48, VM47) melanoma cells as determined by MTT-based cell viability assay. Statistical analysis was done using two-way ANOVA. (**B,C**) Impact of a 72h treatment with Nutlin-3 and YK-4-279 as indicated on viability of (**B**) the melanoma models described in (A), and (**C**) the p53 wild-type, EWS/FLI1-driven FPBH Ewing sarcoma cell model are shown. Raw data (OD) from triplicates are given. Values are given as mean ± SD of one representative out of three experiments performed in triplicate. Statistical significance was tested by Student's t test. p values less than 0.05 were considered significant, with * $p < 0.05$, ** $p < 0.01$, *** $p < 0.001$, **** $p < 0.0001$. (**D**) ETS1 protein levels of two represented melanoma models with intact (VM18) or defective (VM47) p53/21 response after 24h treatment with YK-4-279 at the indicated concentrations as analysed by Western blot.

Figure S6

Dinhof et al.

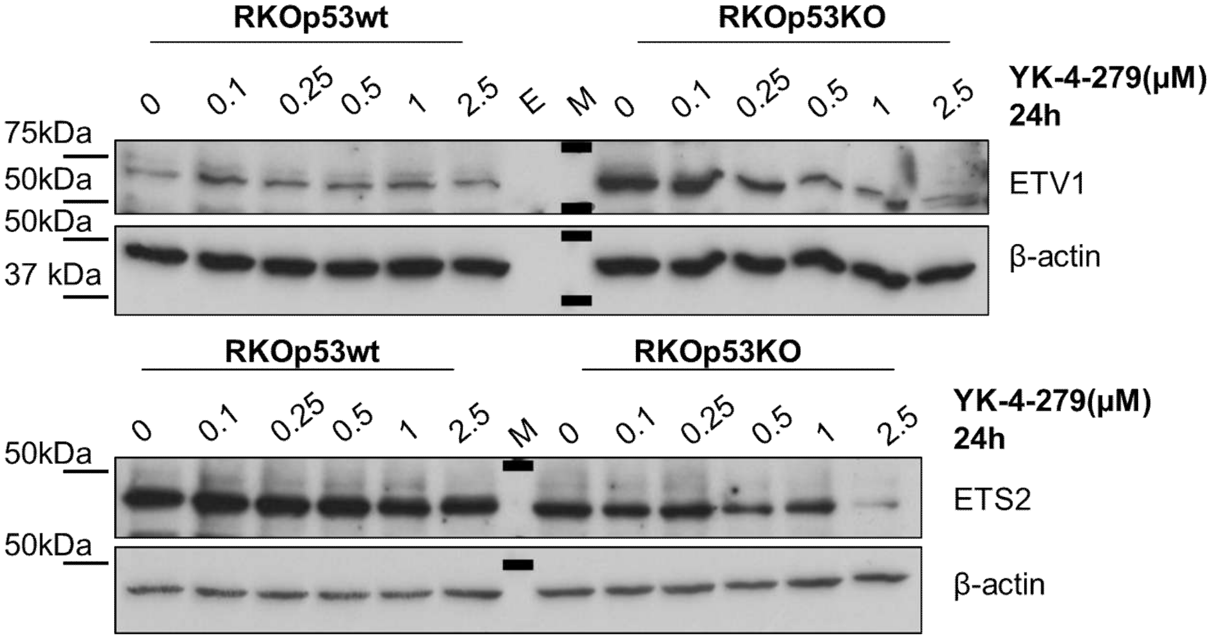

Figure S6: ETV1 and ETS2 protein levels are affected by YK-4-279 in the RKOp53KO model. Effect of 24h incubation with the indicated concentrations of YK-4-279 on protein expression levels of the indicated ETS factors in RKOp53wt and RKOp53KO cells was evaluated by Western blot. β-actin served as loading control. E, empty lane; M, size marker.

Figure S7 **Dinhof et al.**

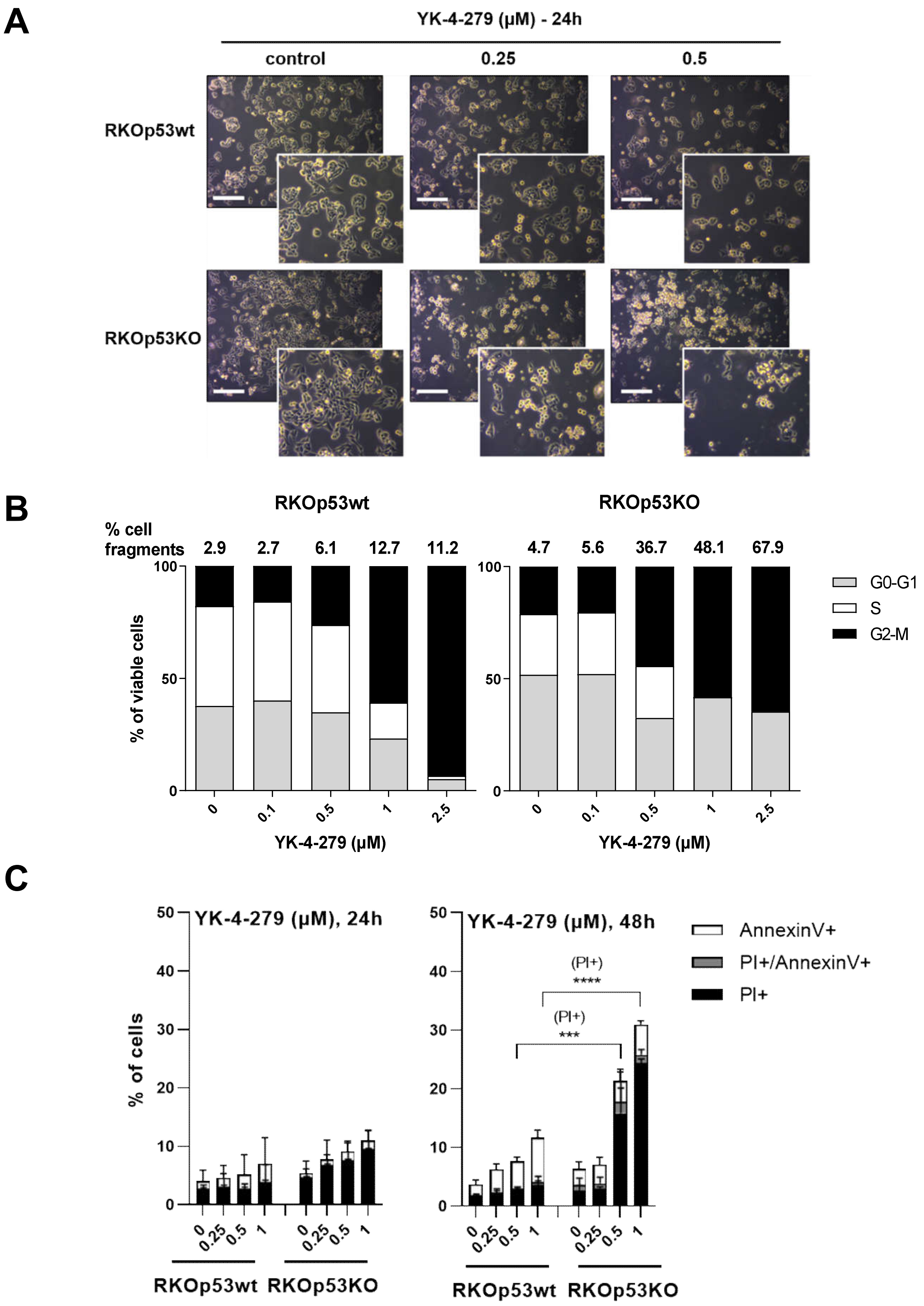

Figure S7: YK-4-279-induced cell death and cell cycle arrest in relation to p53 status of RKO cells. (**A**) Representative photomicrographs of RKO cells treated with the indicated concentrations of YK-4-279 for 24h. Quantification of the analysis is shown in Figure 3D. Scale bar, 500µm. (**B**) Impact of a 24h YK-4-279 treatment at the indicated concentrations on cell cycle distribution (of the viable, non-fragmented cell proportion) are compared to cell fragmentation (given as percentages above the bars). (**C**) Induction of apoptosis and/or necrosis in RKOp53wt compared to RKOp53KO cells by YK-4-279 treatment at the indicated time points was analyzed by Annexin V/PI staining and subsequent flow cytometric analysis. Values are given as % of stained cells ± SD. Two-way ANOVA with Bonferroni post-test was used for statistical testing. p values below <0.05 were considered as significant (*** $p < 0.001$, **** $p < 0.001$).

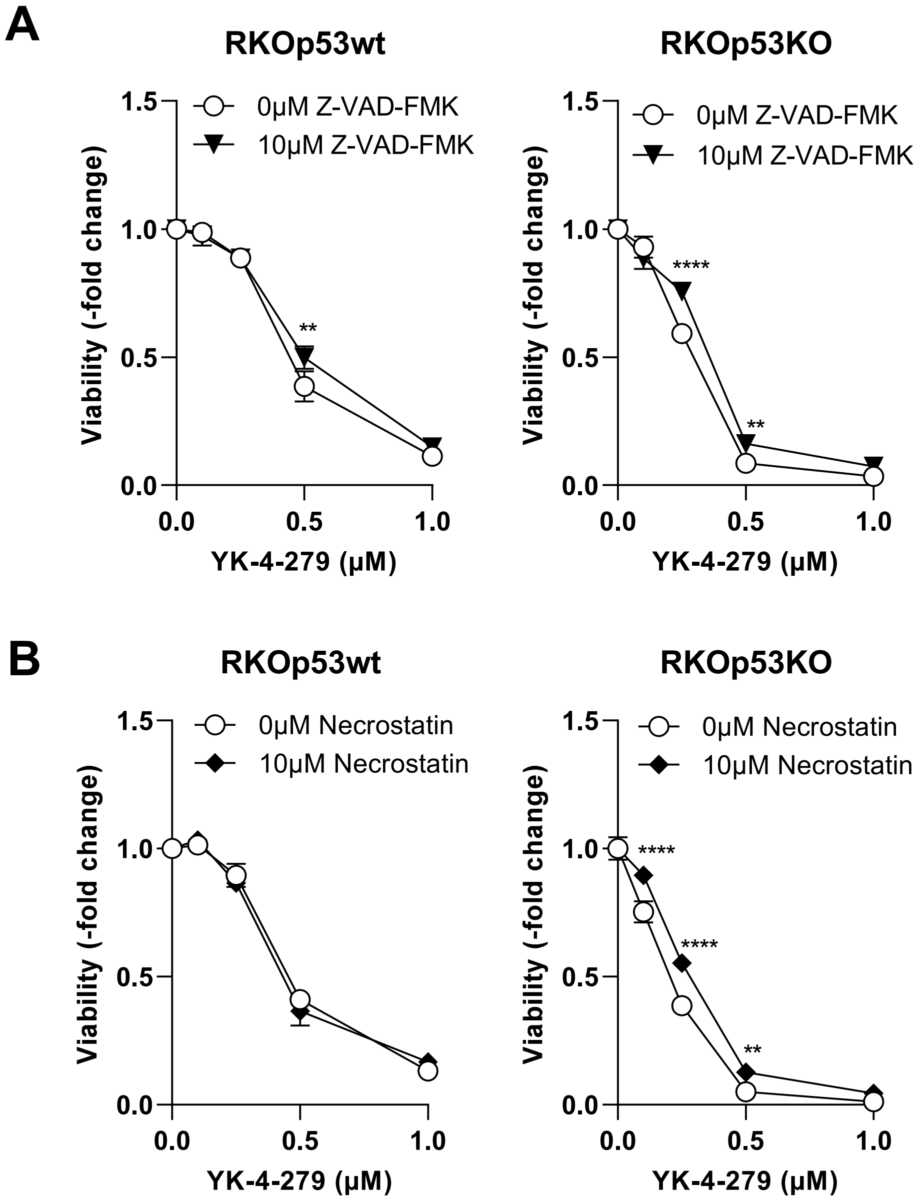

Figure S8: Minor protection by the pan-caspase inhibitor Z-VAD-FMK and the necroptosis inhibitor necrostatin against YK-4-279 cytotoxicity. Effects on viability of RKOp53wt and RKOp53KO cells upon application of either (**A**) Z-VAD-FMK or (**B**) necrostatin in combination with YK-4-279 as indicated for 72h was determined by MTT-based cell viability assay. Values are given as mean ± SD of one representative out of three experiments performed in triplicate. Statistical analysis was done using two-way ANOVA. p values less than 0.05 were considered significant, with ** $p < 0.01$, **** $p < 0.0001$.

Figure S9

Dinhof et al.

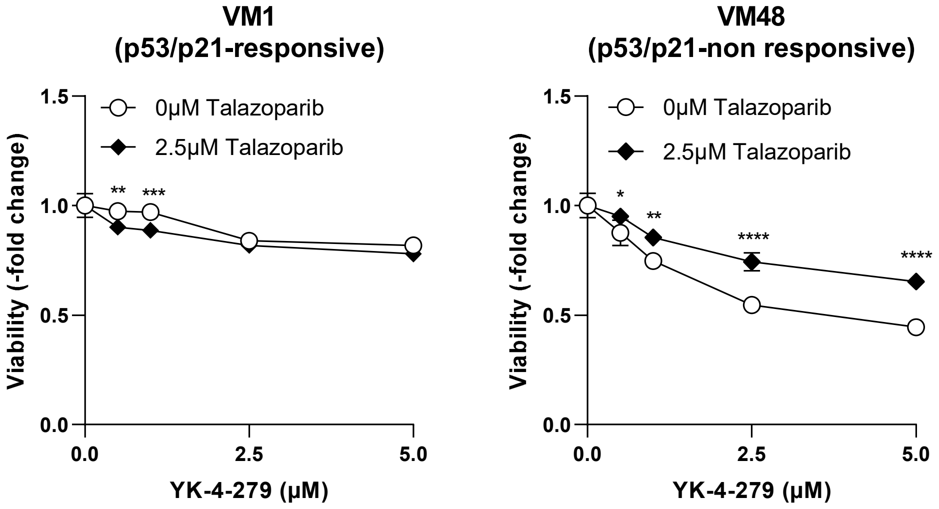

Figure S9: PARP inhibition protects melanoma cells against YK-4-279-induced cytotoxicity. Impact on cell viability of the indicated melanoma models of PARP inhibition by talazoparib in combination with YK-4-279 at the indicated concentrations for 72h was tested by MTT-based assay. Values are given as mean ± SD of one representative out of three experiments performed in triplicate. Statistical analysis was done using two-way ANOVA. p values less than 0.05 were considered significant, with * $p < 0.05$, ** $p < 0.01$, *** $p < 0.001$, **** $p < 0.0001$.
