# Supplementary material for: p53 Loss Mediates Hypersensitivity to ETS Transcription Factor Inhibition Based on PARylation-Mediated Cell Death Induction
Source: Cancers (Basel). 2020 Oct 30;12(11):3205. doi: 10.3390/cancers12113205 (PMC7693367; doi:10.3390/cancers12113205)
Supplement: Supplementary file 1 [file cancers-12-03205-s001.zip › cancers-949334-sup/Table S1_Dinhof et al.pdf]

cancers

Table S1: Antibodies

**Antibody**	**Cat. No./Company**	**Dilution**	**2nd Antibody**
AIF	#5318, Cell Signaling	1:1000	rabbit
BAX	#2772, Cell Signaling	1:1000	rabbit
cleaved PARP1 (Asp214)	#9541, Cell Signaling	1:1000	rabbit
ETS1 (D808A)	#14069, Cell Signaling	1:1000	rabbit
phospho-ETS1 (Thr38)	SAB4503912, Sigma Aldrich	1:500	rabbit
ETS2	sc-365666, Santa Cruz	1:500	mouse
ETV1	H00002115-M01, Abnova	1:250	rabbit
FLI1 (C-19)	sc-356, Santa Cruz	1:1000	rabbit
GAPDH (D16H11)	#5174, Cell Signaling	1:1000	rabbit
Phospho-Histone H2A.X (Ser139)	#9718, Cell Signaling	1:1000	rabbit
Lamin A/C	#4777, Cell Signaling	1:2000	mouse
MCL-1	sc-819, Santa Cruz	1:1000	rabbit
p21	#2947, Cell Signaling	1:1000	rabbit
p53 AB-6 (DO1)	MS-187-P, Thermo Scientific	1:1000	mouse
pADPr	sc-56198, Santa Cruz	1:200	mouse
PARP1	#9542, Cell Signaling	1:1000	rabbit
β-actin	A5441, Sigma Aldrich	1:2000	mouse
mouse-anti-rabbit IgG-HRP	sc-2357, Santa Cruz	1:10000	
mouse IgG-HRP	GTX213111-01, GeneTex	1:10000	
