# Supplementary material for: p53 Loss Mediates Hypersensitivity to ETS Transcription Factor Inhibition Based on PARylation-Mediated Cell Death Induction
Source: Cancers (Basel). 2020 Oct 30;12(11):3205. doi: 10.3390/cancers12113205 (PMC7693367; doi:10.3390/cancers12113205)
Supplement: Supplementary file 1 [file cancers-12-03205-s001.zip › cancers-949334-sup/Table S2_Dinhof et al.pdf]

cancers

Table S2: Primers for qRT-PCR

Gene	Primer forward (sense)	Primer reverse (antisense)
ETS1	5' CTTTCTGACCCAGATGAG 3'	5' GCTGTCTTGTGGATGATG 3'
ETS2	5‘-TCCGTCAGCGTCACCTACT-3‘	5‘-AACCCGTTGCACATCCAG-3‘
ETV1	5′-GCCGTTCACTCCGCTATTAC-3′	5′-CTGTGTCCTCCTCGTTGATG-3’
ETV4	5‘-ACTTCGCCTACGACTCAGATG-3‘	5‘-GAGGTTTCTCATAGCCATAGCC-3‘
ETV5	5‘-CCAAAGATGATGCCTGAAAAC-3‘	5‘-GGGCGATTATCTCCAGGAAC-3‘
RPL-41	5`CAAGTGGAGGAAGAAGCGA 3`	5`TTACTTGGACCTCTGCCTC 3`
